# Designing Biobased Poly(ethylene-*co*-isosorbide terephthalate) Copolyesters with Tunable Properties and Degradability

**DOI:** 10.1021/acs.biomac.4c01630

**Published:** 2025-03-08

**Authors:** Dan Li, Youbing Li, Yu Zhang, Yunsheng Xu, Xianming Zhang, Minna Hakkarainen

**Affiliations:** † School of Materials Science and Engineering, Zhejiang Sci-Tech University, Hangzhou 310018, China; ‡ Zhejiang Provincial Innovation Center Advanced Textile Technology, Shaoxing 312030, China; § Department of Fibre and Polymer Technology, KTH Royal Institute of Technology, Teknikringen 58, Stockholm 10044, Sweden

## Abstract

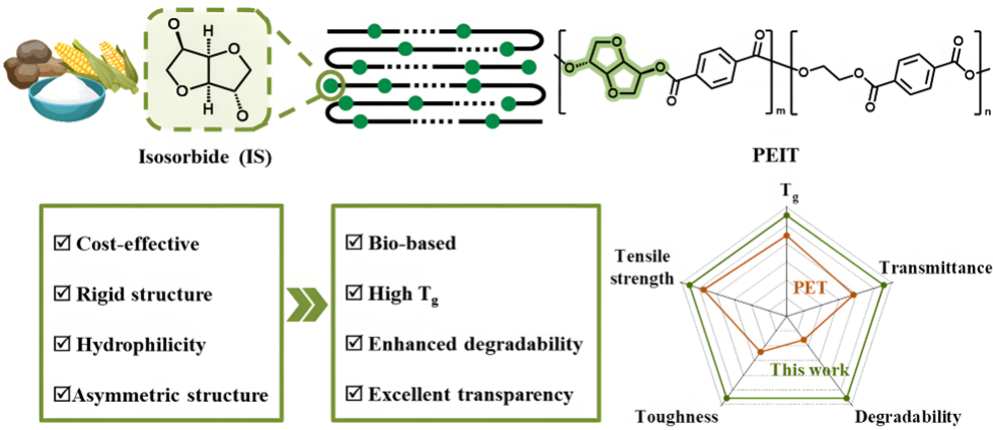

Production of high-performance polyesters with tailored degradability remains a challenge. Here, a series of poly(ethylene-*co*-isosorbide terephthalate) (PEIT) copolyesters were synthesized by varying the isosorbide (IS) content (0–20 mol %) using tetrabutyl titanate (TBT) as the catalyst. By variation of the IS content, the thermal, mechanical, and optical properties of the copolyesters were effectively tailored. As the IS content increased, the *T*_*g*_ was raised from 80 to 101 °C, and the tensile strength from 58.8 to 68.7 MPa. Moreover, excellent transparency was maintained (up to 90% light transmittance). Interestingly, the susceptibility to hydrolytic degradation was significantly enhanced by the incorporation of IS, with PEIT-20 showing approximately 3.5 times higher weight loss compared to PET after 50 days of alkaline degradation in 0.1 M NaOH solution. This outlines an attractive approach for developing high-performance copolyesters with tunable properties and degradation rates, suitable for applications in transparent thermal packaging materials.

## Introduction

Plastics have become integral to industry, agriculture, and everyday life due to their versatile and customizable properties.^1,2^ However, the majority of plastics currently in use are derived from nonrenewable fossil resources and are highly resistant to chemical and biological degradation.^3−5^ This reliance on nonrenewable feedstocks not only contributes to increasing CO_2_ emissions but also leads to significant environmental issues, such as the accumulation of nondegradable plastic waste and microplastics.^6−11^ Consequently, the development of biobased and/or degradable plastics has emerged as one vital strategy to address these environmental challenges.

Polyesters occupy a significant role in the field of degradable plastics due to the unique degradation sites provided by their ester bonds.^12,13^ Most commercially available degradable polyesters are aliphatic in nature, including poly(lactic acid) (PLA), poly(3-hydroxybutyrate) (PHB), and poly(butylene succinate) (PBS). However, combining degradability with good heat resistance and mechanical properties in these materials remains a significant challenge.^14,15^ Poly(butylene adipate-*co*-terephthalate) (PBAT) as a commercially available semiaromatic polyester has become a valued complement to the aliphatic polyesters due to its favorable ductility, processing characteristics, and degradability. The incorporation of aliphatic adipate units to replace some of the terephthalate units provides degradation-susceptible links and enhances the degradability of PBAT. Despite these advantages, the increased aliphatic content also leads to a lower glass transition temperature (*T*_*g*_, ranging from −30 to −15 °C), which limits the use in engineering applications.^16,17^ Therefore, the development of a polyester that can achieve a balance between high thermal and mechanical performance while maintaining adequate degradability remains a significant challenge.

Isosorbide (IS), a rigid bicyclic diol derived from renewable resources such as starch and cellulose, has emerged as a promising biobased monomer.^18^ It has been widely investigated for the synthesis of a variety of polymers, including polycarbonates,^19−21^ polyurethanes,^22−24^ polysulfones,^25,26^ polyamides,^27−29^ polyethers,^30,31^ and polyesters.^32−35^ Due to its unique bicyclic ring structure and aliphatic nature, incorporating IS into polyester chain can enhance both thermal and mechanical properties, but it could also promote the susceptibility to degradation.^36,37^ Specifically, IS can improve the performance of aliphatic polyesters by addressing common limitations such as inadequate heat resistance and low tensile strength while providing tailorable crystallinity and barrier properties. Despite these advantages, most reported IS-based aliphatic polyesters still exhibit relatively low glass transition temperatures, typically ranging from −25 to 5 °C.^38−41^ To overcome this, recent research has focused on copolymerizing the IS with aromatic monomers. For instance, Legrand et al.^32^ developed an amorphous IS-based copolyester with a significantly higher *T*_*g*_ (115 to 163 °C) and excellent heat deflection properties. Similarly, Chen et al.^42^ demonstrated that copolymerizing IS with poly(butylene terephthalate) (PBT) greatly increased the *T*_*g*_ and modulus of the resulting copolyesters, while it simultaneously enhanced the hydrolytic degradation rate under acidic conditions. Poly(ethylene terephthalate) (PET) has higher *T*_*g*_ and wider industrial application range compared to PBT. This makes incorporation of IS into PET chain to yield poly(ethylene-*co*-isosorbide terephthalate) (PEIT) copolyesters an interesting option for achieving improved thermal and mechanical properties.^43^ At the same time, we hypothesize that the susceptibility to degradation could also be tunable by the IS content.

Synthesis of PEIT was reported previously primarily through direct esterification processes. IS as a secondary diol has lower reactivity than ethylene glycol and in addition its endohydroxyl groups form intramolecular hydrogen bonding with the adjacent furanic oxygen causing different reactivities for the two hydroxyl groups.^44−46^ Esterification typically requires high temperatures to overcome the substantial activation energy of the reaction. Unfortunately, IS is prone to ring-opening side reactions at elevated temperatures. Furthermore, as IS exhibits limited reactivity during the esterification/transesterification stages, it is easily lost during the vacuum polycondensation stage, making it difficult to control the IS content in the final copolyester product. To address these issues, organic and metal catalysts for synthesizing PEIT have been researched. Stanley et al.^47^ compared the catalytic efficiency of several organic catalysts in the polycondensation stage of PEIT, showing that the achieved molecular weight is somewhat lower compared with the molecular weight achieved with metal catalysts. Research on metal catalysts often focused on the development of bimetallic catalyst systems by the incorporation of additional metals into commercial catalysts commonly used for PET synthesis.^43,48−50^ There is, however, a lack of systematic screening of different types of catalysts and comprehensive studies on the overall performance of such copolyesters, including their degradation behavior.

Previous research on PEIT has primarily focused on the synthesis challenges posed by IS, particularly its low reactivity and susceptibility to side reactions during high-temperature processes. To address these issues, we systematically investigated several organic and metal catalysts to enhance the reactivity and retention of IS during the transesterification stage. Furthermore, by varying the IS content, we demonstrate how PEIT copolyesters exhibit a balance between high thermal and mechanical performance and tunable degradation rates. Comprehensive analyses of catalytic efficiency, molecular weight, and degradation behavior provide valuable insights into the structure–property relationships of these copolyesters. This advances the understanding of IS-based copolyesters and contributes to the development of high-performance biobased materials with broad application potential.

## Experimental Section

### Materials

Dimethyl terephthalate (DMT, 99%), ethylene glycol (EG, 99%), isosorbide (IS, 98%), tetrabutyl titanate (TBT, 99%), trifluoroacetic acid (TFA, 99%), ethyl acetate (99%), hexafluoroisopropanol (HFIP, 99.5%), 1,1,2,2-tetrachloroethane (98%), and phenol (99%) were purchased from Aladdin Chemical Reagent (Shanghai, China). Chloroform (99%) was obtained from Sinopharm Chemical Reagent (Shanghai, China). Methanol (99.5%) was received from Shanghai Zhanyun Chemical Co., Ltd. All chemicals above were used as received except for IS, which was further purified by recrystallization from ethyl acetate before use.

### Synthesis of Poly(ethylene-*co*-isosorbide terephthalate) (PEIT)

PEIT copolyesters with different compositions were synthesized from DMT, EG, and IS through a two-step melt polycondensation (transesterification and polycondensation) procedure. Tetrabutyl titanate (400 ppm relative to the total monomer mass) was added to the reactor as a catalyst. The synthesized copolymers are named PEIT-*x*, where *x* represents the molar percentage of IS relative to the total diol amount in the feed. The reaction was performed in a 500 mL three-neck round-bottom flask equipped with a mechanical stirrer with torque measurement, a nitrogen gas inlet, and a reflux condenser connected with a decompressor.

In the first step, DMT (0.4 mol, 77.7 g, 1.0 equiv), EG, IS, and TBT (400 ppm) were added to the flask. The feed molar ratio of diols to diesters was fixed to 1.5:1. The reaction was conducted at 220 °C for 3–4 h under a constant nitrogen flow with 120 rpm stirring. In the second step, the reaction temperature was stepwise raised to 270 °C and the pressure was reduced to 60 Pa in 60 min. The stirring speed was decreased slowly to 20 rpm. After 4 h, the crude product was obtained. It was dissolved in a mixture of CHCl_3_/TFA (6:1 v/v), and the solution was subsequently added dropwise into an excess of methanol. The precipitated polymer was filtered from methanol and dried at 120 °C under vacuum to a constant weight.

### Characterization

Intrinsic viscosities (η) of the copolyesters were measured in a solvent mixture consisting of phenol and 1,1,2,2-tetrachloroethane (1:1 w/w) by an IVS400-2 Ubbelohde viscometer at 25 ± 0.1 °C. The samples were prepared with a concentration of 0.25 g/dL. Fourier transform infrared (FT-IR) spectra of the copolyesters were recorded using a Nicolet iS50 FT-IR spectrometer, which was equipped with an attenuated total reflectance (ATR) accessory. All the spectra were scanned in the range of 4000–400 cm^–1^ with 32 scans. The ^1^H nuclear magnetic resonance (^1^H NMR) spectra were recorded on a Bruker Avance III HD 400 MHz liquid-state spectrometer, and tetramethylsilane (TMS) was used as an internal standard. Deuterated trifluoroacetic acid was used as a solvent. The number-average molecular weight (*M*_*n*_), weight-average molecular weight (*M*_*w*_), and dispersity (*Đ*) of the copolyesters were determined using a Waters ACQUITY Advanced Polymer Chromatography (APC) coupled with multiangle laser light scattering (MALLS) detection at 55 °C. HFIP was used as a solvent and mobile phase at a flow rate of 0.4 mL/min. The sample concentrations were controlled at approximately 2 mg/mL. The injection volume per sample was 30 μL. The frequency sweep measurements were performed on an Anton Paar MCR 702e. The experiments were conducted in a convection temperature device at 265 °C with a strain of 1%. The frequency was varied from 100 to 0.1 rad·s^–1^.

Thermogravimetric analysis (TGA) was evaluated by using a NETZSCH STA 2500 Regulus. All of the samples (6–8 mg) were heated from 25 to 600 °C under a nitrogen flow of 40 mL/min at a heating rate of 10 °C/min. Differential scanning calorimetry (DSC) analysis was performed on a METTLER-TOLEDO DSC 3 instrument under a constant nitrogen flow of 40 mL/min. All of the samples (6–8 mg) were first heated from 25 to 300 °C, maintained at 300 °C for 2 min, then cooled to 25 °C and kept at 25 °C for 2 min, before reheating to 300 °C. Both heating and cooling rates were set to 10 °C/min. Wide-angle X-ray diffraction (WAXD) patterns were recorded with an Empyrean X-ray diffraction instrument equipped with a Cu Kα source (λ = 0.154 nm). The scanning range was 5–90° and the step was 0.02°. Dynamic mechanical analysis (DMA) was conducted in tension film mode on a METTLER TOLEDO dynamic mechanical analyzer 1 using rectangular film specimens (25 × 6 × 0.2 mm^3^). All the measurements were conducted in the temperature range from 25 to 200 °C with a heating rate of 5 °C/min. The frequency was set to 10 Hz. Dumbbell-shaped samples (about 40 × 5 × 2 mm^3^ in length, width, and thickness, respectively) for tensile testing were prepared by injection molding at 240–280 °C, with an injection pressure of 0.4 MPa, and a mold temperature of 55 °C. Before the tests, all the samples were conditioned at 22 °C and 40% relative humidity for 72 h. The tensile properties of each sample were calculated from the average of five repeated measurements. The tensile test was determined on an Instron 34TM-30 universal testing machine at room temperature, and the crosshead speed was 5 mm/min.

The film samples were prepared by hot compression molding using a heat press at a temperature within the range of 230–280 °C (20 °C above the melting point of each copolyester), followed by pressing at 10 MPa for 3–5 min. The surface morphology of the degraded samples and tensile-fractured surfaces was characterized by a ZEISS Sigma500 field emission scanning electron microscope (SEM). The surface of samples was spray coated with a gold film prior to the SEM measurement. Ultraviolet–visible (UV–vis) optical transmission spectra of the copolyester films (80 × 80 × 0.2 mm^3^) were measured using a HITACHI UH4150 spectrophotometer within the wavelength range of 200–800 nm. The CIE color parameters of the copolyester films (80 × 80 × 0.2 mm^3^) were conducted on an SF-300 colorimeter (Datacolor, USA). The N_2_ and O_2_ barrier properties of the copolyesters were measured with a Labthink VAC-V2 gas permeability tester at 23 °C and 50% RH, while the water vapor barrier property was studied with the Labthink W3-060 water permeability tester at 38 °C and 90% RH. All gas barrier tests were conducted on copolyester films with dimensions of 100 × 100 × 0.2 mm^3^. To ensure the reproducibility of the results obtained, three independent specimens were tested for each sample. The hydrophilicity of the copolyester films (80 × 80 × 0.2 mm^3^) was tested by a KRUSS DSA25 contact angle meter. Water (3 μL) was dropped onto the sample surface, and the photo was taken immediately. At least five drops were observed in different areas for each film, and their average values were obtained.

Hydrolysis degradation experiments of the copolyester films with a thickness of 0.2 mm were carried out in a 0.1 M NaOH solution at 58 °C. Before the tests, all of the films were dried under vacuum for 24 h at 120 °C. The ratio between the amount of NaOH solution (g) and the mass of the dried sample (g) was set at 550. The NaOH solution was not replaced during the hydrolysis experiment. One film was taken out after each predetermined time, thoroughly washed with distilled water, and dried under vacuum to constant weight. The degree of degradation was estimated from the mass loss (*D*) according to eq 1.
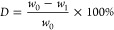
1where *w*_0_ is the original weight, and *w*_1_ is the residual weight after degradation.

## Results and Discussion

### Catalyst Screening

**Scheme 1:**
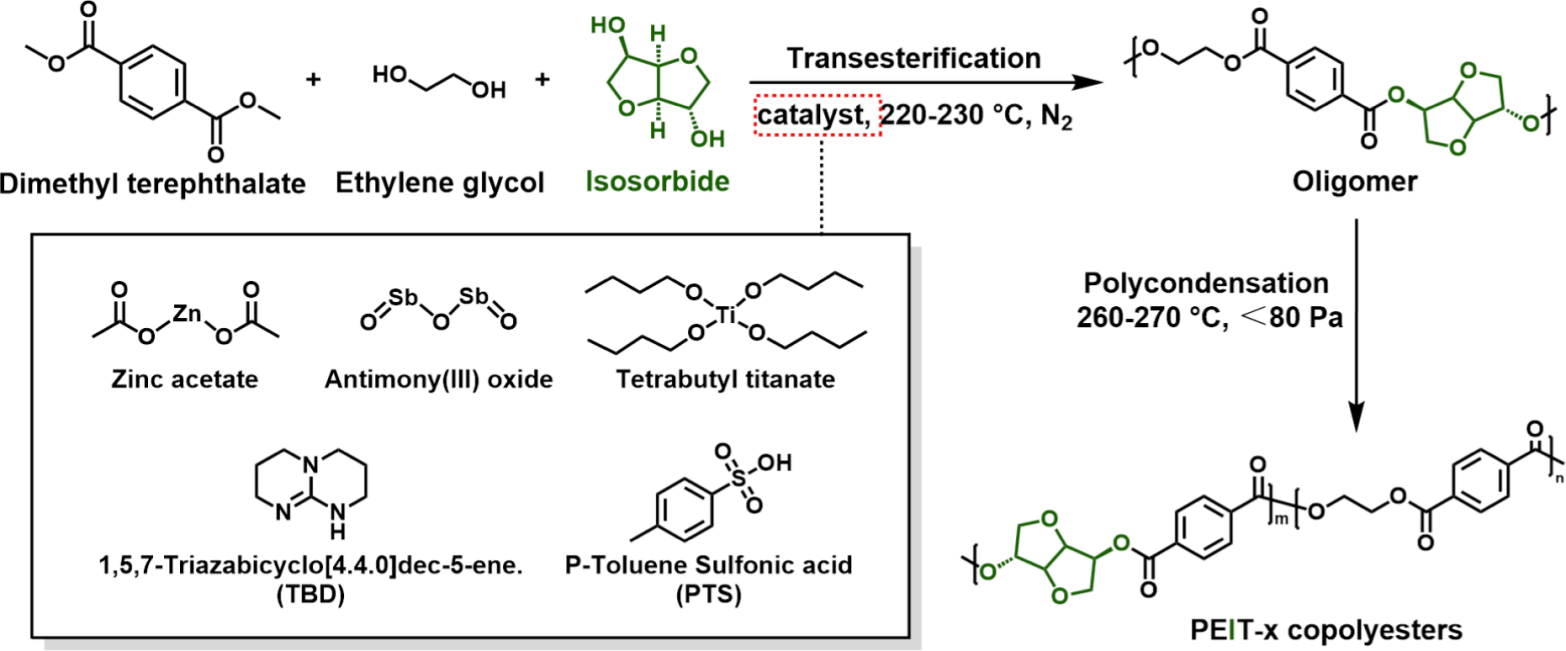
Synthesis Route of PEIT Copolyesters

Isosorbide (IS) was selected as a rigid biobased monomer to synthesize a series of copolyesters with dimethyl terephthalate (DMT) and ethylene glycol (EG). Unreacted IS is easily removed during the vacuum polycondensation stage, leading to a low proportion of IS structural units and a reduced molecular weight in the final copolyester product. Therefore, the conversion rate of IS during the transesterification stage significantly affects the final molecular weight and monomer conversion rate. Five common catalysts were evaluated to specifically investigate the conversion rate of IS during the transesterification stage: three metal catalysts (Zn(Ac)_2_, Sb_2_O_3_, and TBT), one organic acid catalyst (PTS), and one organic base catalyst (TBD) (Scheme 1).

**Figure 1:**
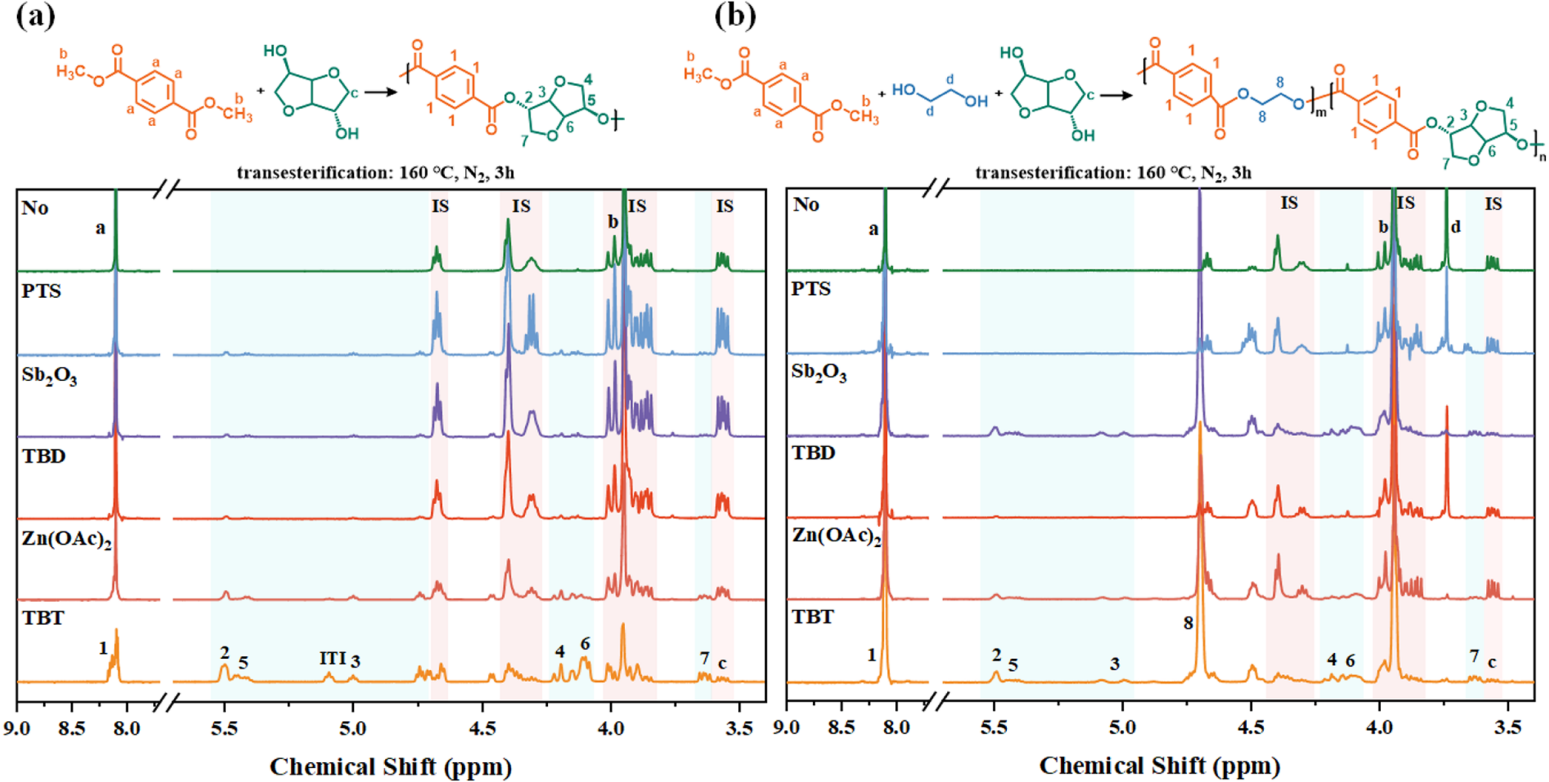
^1^H NMR spectra of oligomers from transesterifications a) between DMT and IS and b) between DMT, EG, and IS with different catalysts.

Due to the low hydroxyl reactivity and relatively low boiling point of IS, its transesterification reaction with DMT is very low in the absence of catalysts. The structure of the oligomers produced by the transesterification reaction under different catalysts was studied using ^1^H NMR (Figure 1). The monomer conversion rates were calculated from the area of characteristic peaks in ^1^H NMR and are presented in Table 1.

**Table 1:** Monomer Conversion Rates during Transesterification Reactions with Different Catalysts

			IS conv.^a^ (%)		
Catalyst type	Catalyst	Temperature (°C)	Two-phase	Three-phase	EG conv.^b^ (%)
-	No cat.	160	0	0	0
Metal	Zn(OAc)_2_	160	27	12	99
Metal	Sb_2_O_3_	160	2	76	99
Metal	TBT	160	70	97	99
Organic base	TBD	160	2	18	15
Organic acid	PTS	160	1	0	11

a . Where peak c corresponds to the proton on the IS monomer before the transesterification reaction, and peak 7 corresponds to the proton on the IS unit in the oligomers after the transesterification reaction.

b . Where peak d corresponds to the proton on the EG monomer before the transesterification reaction, and peak 8 corresponds to the proton on the EG unit in the oligomers after the transesterification reaction.

The biphasic system of DMT and IS was first studied under a nitrogen atmosphere at 160 °C for 3 h to conduct the transesterification reaction. Figure 1a shows that the red-shaded peaks represent unreacted IS, while the blue-shaded peaks indicate IS units in the oligomers. The IS conversion rate was calculated from the peaks in the 3.5–3.7 ppm range. The catalytic efficiency of the five catalysts for IS follows the order: TBT > Zn(Ac)_2_ > TBD > Sb_2_O_3_ > PTS. TBT exhibited the highest IS conversion rate at 70%.

Subsequently, the catalytic efficiency of the selected catalysts was investigated in the triphasic system of DMT, IS, and EG (IS accounting for 15% of the total diol content). As shown in Table 1, the addition of EG increased the IS conversion rates. The main reasons are as follows: first, EG acts both as a monomer and as an effective mass transfer solvent, improving the dispersion and solubility of the catalyst in the system. It facilitates better contact between the active sites of the catalyst and the monomers, thereby increasing the catalytic efficiency. As an example, Sb_2_O_3_ has poor solubility and generally functions as a heterogeneous catalyst. In the biphasic system, it catalyzed the IS conversion rate to only 2%, whereas its catalytic efficiency increased to 76% in the ternary system. Second, EG, as a primary diol, preferentially reacts with DMT and can be regarded as a “chain connector”.^51^ Moreover, the IS content was reduced compared with the biphasic system, with most IS units present as end groups in the oligomers. In the ternary system, TBT maintained the highest IS conversion rate, reaching 97%. Besides, the EG conversion rate was calculated from the peaks at 3.9 and 4.7 ppm in Figure 1b, revealing that the three metal catalysts all maintained EG conversion rates of 99%. Based on these findings, TBT was selected as the catalyst for the synthesis of PEIT copolyesters.

### Synthesis and Structural Characterization of PEIT Copolyesters

**Figure 2:**
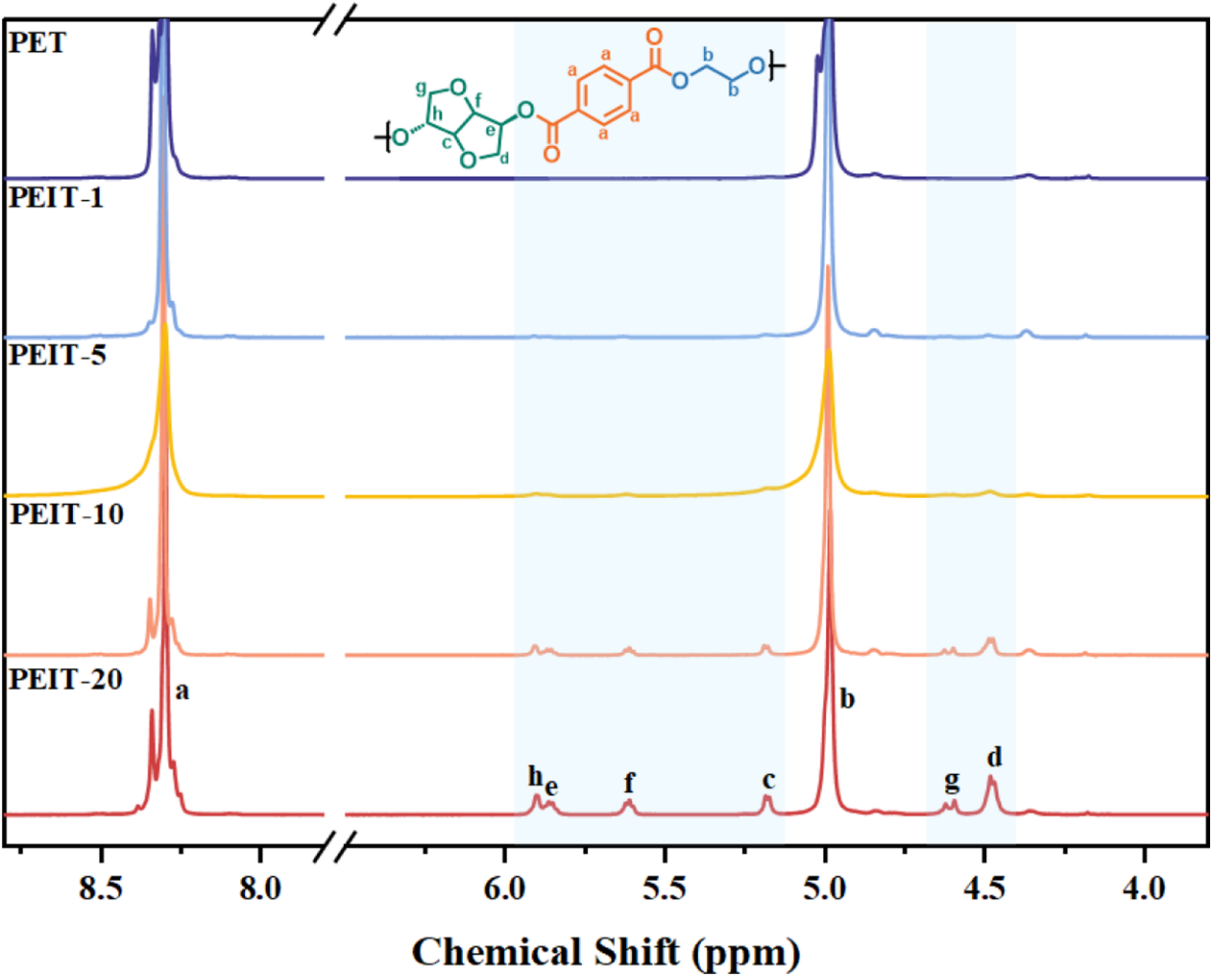
^1^H NMR spectra of PEIT copolyesters with different IS contents.

PEIT copolyesters with varying IS contents (0, 1, 5, 10, and 20% of the total diol content) were successfully synthesized through a two-step transesterification melt polycondensation. The chemical composition of the copolyesters was initially examined by FTIR (Figure S1), revealing no significant structural differences compared to PET. The chemical structure of PEIT copolyesters with different IS contents was further investigated by ^1^H NMR spectroscopy (Figures 2and S2-3). The new peaks in the shaded regions correspond to hydrogen atoms in the IS structural units, confirming the successful synthesis of the PEIT-*x* copolyesters.

**Figure 3:**
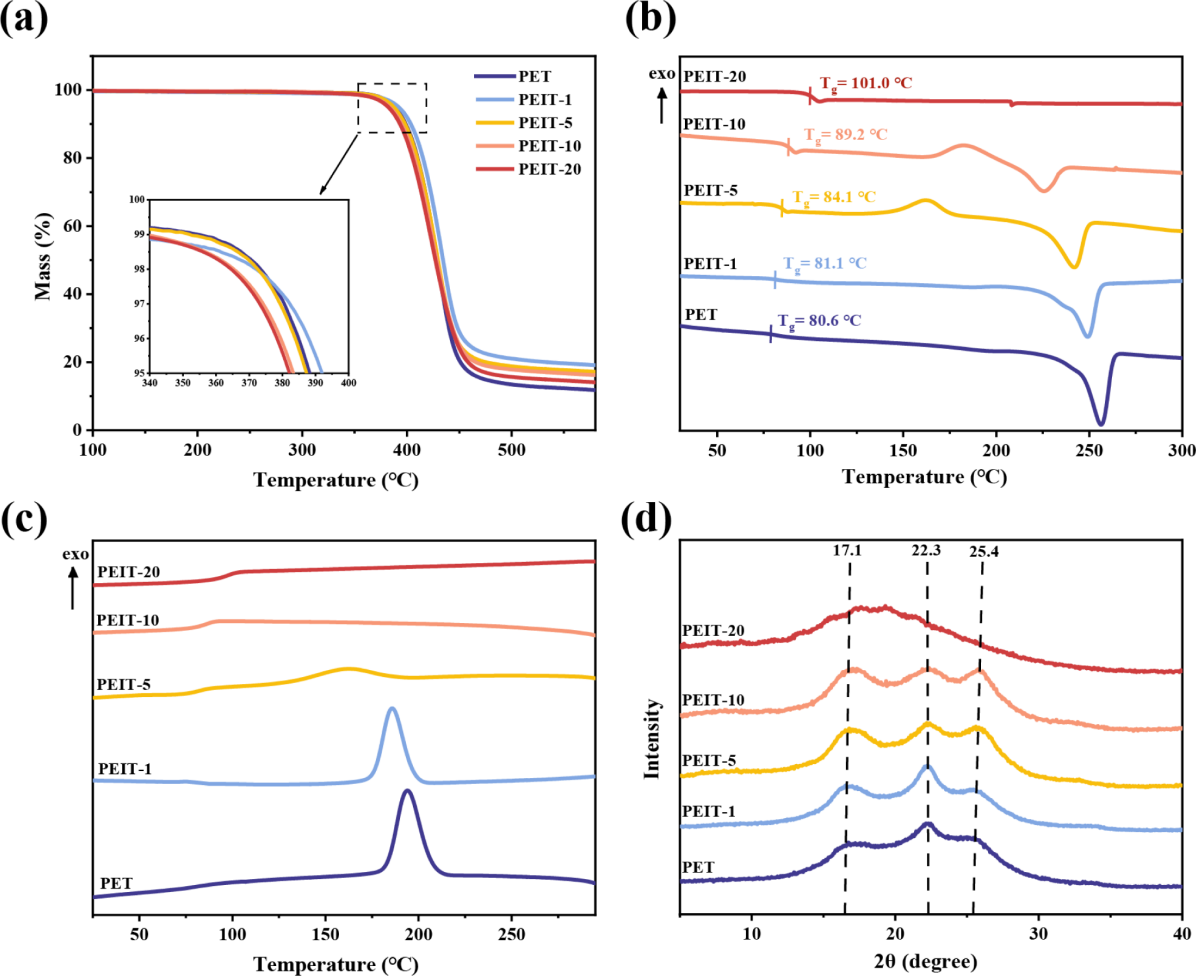
Thermal and crystal properties of PEIT copolyesters with different IS contents. (a) TGA curves. (b) The second heating DSC curves. (c) First cooling DSC curves. (d) Wide angle X-ray diffraction patterns.

The feed ratios, incorporation ratios, intrinsic viscosities, molecular weights, and dispersities of the copolyesters are summarized in Table 2. The actual molar contents of IS units in copolyesters were calculated from the integration ratio of peaks c and b in Figure 2. The results closely matched the feed ratios, indicating that both the primary and secondary hydroxyl groups were effectively activated by TBT during the transesterification stage. The intrinsic viscosities of the copolyesters, measured by using an automatic viscometer, ranged from 0.64 to 0.86 dL/g. As the IS content increased, the intrinsic viscosity of the copolyesters decreased. However, all copolyesters maintained an intrinsic viscosity above 0.6 dL/g, meeting the general requirements for fiber-grade industrial polyesters.^52^

**Table 2:** Chemical Composition, Intrinsic Viscosity, and Molecular Weight of PEIT Copolyesters with Different Isosorbide Contents

Sample	Feed ratio	Incorporation ratio^a^	η^b^ (dL/g)	*M*_*n*_^c^ (g/mol)	*M*_*w*_^c^ (g/mol)	*Đ*^c^ (*M*_*w*_ /*M*_*n*_)
PET	0/100	0/100	0.83	28,800	42,000	1.5
PEIT-1	1/99	1.2/98.8	0.86	38,900	56,600	1.5
PEIT-5	5/95	4.9/95.1	0.68	22,200	35,700	1.6
PEIT-10	10/90	10.6/89.4	0.66	19,600	37,100	1.9
PEIT-20	20/80	19.6/80.4	0.64	18,000	34,400	1.9

a Incorporation molar ratios were determined by ^1^H NMR spectroscopy.

b Intrinsic viscosity was measured in a mixed solvent of phenol and 1,1,2,2-tetrachloroethane (50/50 w/w) at 25 °C.

c Molecular weights and dispersities were characterized by APC in HFIP.

The absolute molecular weights of the copolyesters were measured by APC (Figure S4). The *M*_*n*_ ranged from 18,000 to 40,060 g/mol and *M*_*w*_ from 34,440 to 47,930 g/mol, while *Đ* was between 1.5 and 1.9. As expected, the molecular weight of the copolyesters generally decreased with increasing IS content, and the dispersity broadened due to the steric hindrance of IS leading to lower reactivity during the polycondensation stage. However, the molecular weight of the copolyesters increased significantly when 1% of the total diol molar content was IS. Therefore, we further investigated the transesterification product during the synthesis of PEIT-1 through a frequency sweep test and differential scanning calorimetry test (Figures S5–S6). It was found that the complex viscosity of the transesterification product of PEIT-1 was much higher compared with the other compositions at 265 °C and already exhibited comparable thermal properties to the final polycondensation product. In contrast, the transesterification products of other compositions melted completely below 200 °C. These results indicate that the transesterification rate increases with a small amount of IS. Thereby, it seems that IS not only acts as a third monomer but also exhibits self-catalytic behavior.

### Thermal Properties and Crystal Structure

The thermal stability of all copolyesters was investigated by TGA, as shown in Figure 3a and Table 3. All copolyesters underwent a single-stage thermal degradation and demonstrated high thermal stability since all of the samples exhibited *T*_d-5%_ above 380 °C. The residue weights at 600 °C were between 12 and 22%. Likely, due to the higher molecular weight of PEIT-1, its *T*_d-5%_ was slightly higher than that of PET. With a further increase in IS content, the *T*_d-5%_ of the copolyesters gradually decreased, although the differences were not large. This was attributed to the decreasing molecular weight of the copolyesters. The thermal decomposition temperature of IS monomer was only 270 °C.

**Table 3:** Thermal Properties of PEIT Copolyesters with Different Isosorbide Contents

Sample	*T*_d-5%_^a^ (°C)	*R*_*w*_^b^ (%)	*T*_*g*_^c^ (°C)	*T*_*m*_^d^ (°C)	Δ*H*_*m*_^d^ (J/g)	*T*_*c*_^e^ (°C)	*ΔH*_*c*_^e^ (J/g)	*T*_*cc*_^f^ (°C)	*ΔH*_*cc*_^f^ (J/g)	*X*_*c*_^g^ (%)
PET	388	12	80	256	44	194	42	-	-	30
PEIT-1	391	22	81	249	33	186	36	-	-	26
PEIT-5	387	17	84	242	28	162	7	162	8	11
PEIT-10	383	16	89	226	12	-	-	182	7	5
PEIT-20	382	14	101	208	1	-	-	-	-	-

a Temperature at which 5% weight loss was observed in the TGA traces.

b Remaining weight at 600 °C.

c Glass transition temperature (*T*_*g*_) was taken as the inflection point of the second heating DSC traces.

d Melting points (*T*_*m*_) and their respective enthalpies (Δ*H*_*m*_) were measured from the second DSC heating curves.

e Crystallization temperatures (*T*_*c*_) and their respective enthalpies (Δ*H*_*c*_) were measured from the first DSC cooling curves.

f Cold crystallization temperatures (*T*_*cc*_) and respective enthalpies *(*Δ*H*_*cc*_) were measured from the second DSC heating curves.

g Degree of crystallinity was calculated by dividing Δ*H*_*cc*_ with the enthalpy (Δ*Η*_*C*_*°* = 138 J/g) of 100% crystalline PET.

The thermal properties of the copolyesters were explored by DSC. The glass transition temperature (*T*_*g*_), melting temperature (*T*_*m*_), crystallization temperature (*T*_*c*_), cold crystallization temperature (*T*_*cc*_), and corresponding enthalpies are listed in Table 3. The DSC curves are shown in Figure 3b,c. The *T*_*g*_ significantly rose from 80 to 101 °C with increased IS content, which is higher than values typically reported for furan-based copolyesters (70–80 °C)^53,54^ and 1,4-cyclohexanedimethanol (CHDM)-based copolyesters (75–90 °C).^55,56^ This increase can be attributed to the rigid bicyclic structure of the IS, which restricts chain segmental mobility and increases the energy required for molecular motion. In contrast, *T*_*m*_ decreased from 256 to 208 °C, thereby reducing the required processing temperature, which can otherwise be an issue for PET. Meanwhile, the crystallinity of the copolyesters gradually decreased as a function of IS content. When 20 mol % of EG was replaced by IS, PEIT copolyesters transitioned from semicrystalline to amorphous materials. This is due to the steric hindrance introduced by IS, which disrupts the regular packing of polymer chains and reduces the ability to form crystalline domains. A similar trend has been reported for poly(butylene terephthalate-*co*-isosorbide terephthalate) (PBIT) copolyesters,^42^ where the inclusion of IS units led to increased *T*_*g*_ (from 31 to 91 °C) and decreased crystallinity (from 39 to 0%). However, PBIT still exhibited some crystallinity, even when the IS content exceeded 30%. This is explained by the longer aliphatic chain segment of butylene glycol in comparison to that of ethylene glycol, which increases the chain mobility. Furthermore, having two diols with more equal lengths could increase the regularity of the PBIT chain, thus promoting crystallization.

The crystal structure of PEIT copolyesters was further studied by using WAXD, and the diffraction curves are shown in Figure 3d. The diffraction patterns of copolyesters containing 1–10 mol % IS exhibited peak positions identical to those of PET homopolyester. The diffraction peaks appeared at 2θ angles of 17.1°, 22.3°, and 25.4°, corresponding to the (100), (110), and (111) crystal planes, respectively. This indicates that the crystal structure of the copolyesters was not altered by the introduction of IS, suggesting that IS units primarily reside in the amorphous regions of the copolyesters. Additionally, the intensity of these diffraction peaks weakened with increasing IS content, consistent with our DSC findings.

### Mechanical Properties

**Figure 4:**
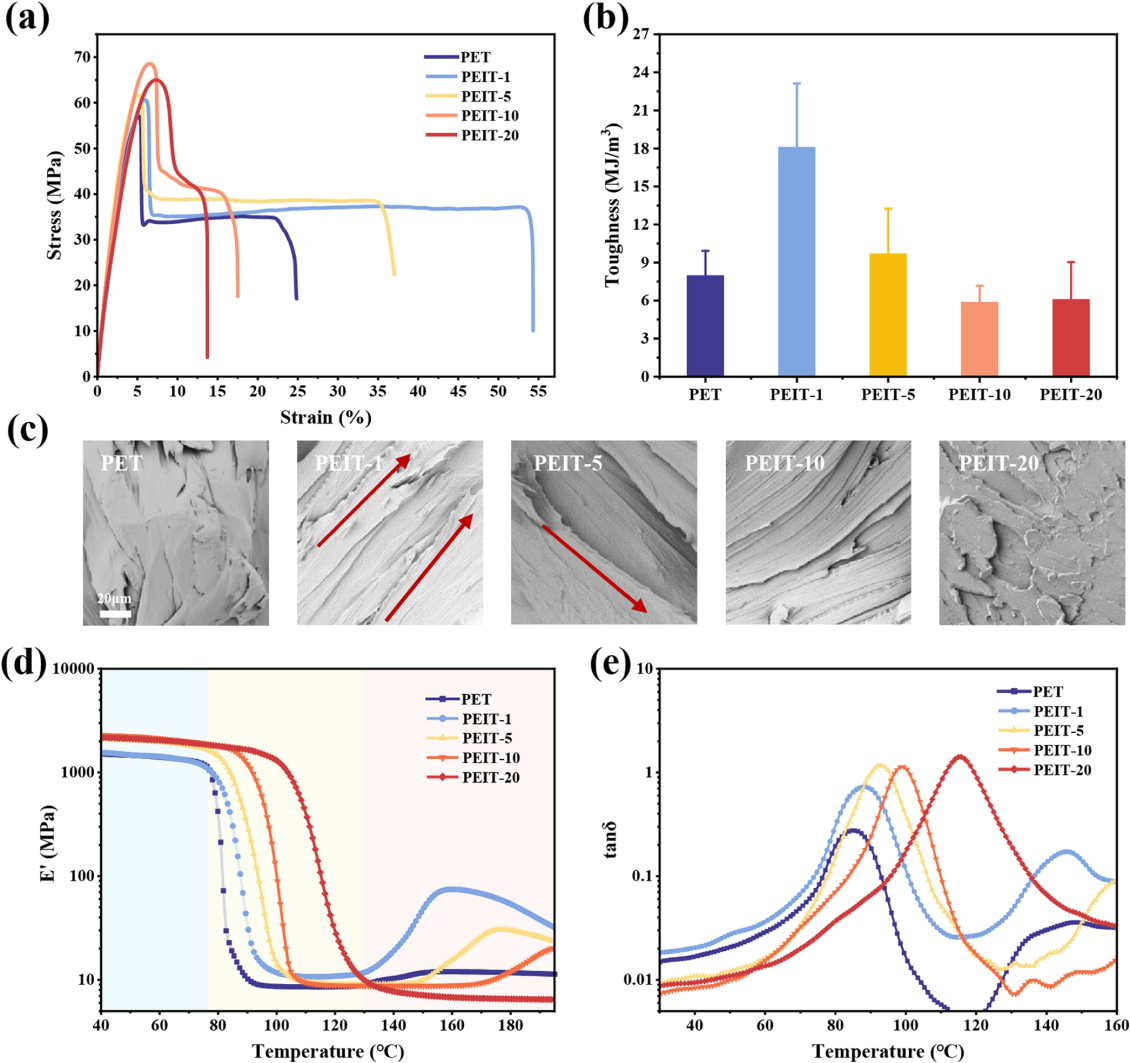
Mechanical properties and dynamic thermal–mechanical properties of PEIT copolyesters with different IS contents. (a) Tensile stress–strain curves. (b) Toughness (*U*). (c) SEM images of the fractured surface after tensile test. (e) Storage modulus (*E*’). (f) Loss factor (tan δ).

The mechanical properties of PEIT copolyesters were examined through tensile testing, with stress–strain curves and toughness shown in Figure 4a,b, and the corresponding data are displayed in Table 4. The introduction of rigid IS units significantly increased the tensile strength of PEIT copolyesters. With 10% IS, tensile strength increased by about 10 MPa. However, at 20% IS, tensile strength slightly decreased and the material exhibited brittle fracture.

**Table 4:** Mechanical Properties of PEIT Copolyesters with Different Isosorbide Contents

Sample	σ (MPa)	*E* (MPa)	ε (%)	*U*^a^ (MJ/m^3^)	*T*_*g*_^b^ (°C)	*E’*^c^ (MPa)
PET	58.8 ± 3.0	1740 ± 83	22 ± 5	8.0 ± 1.9	85	1540
PEIT-1	59.5 ± 1.5	1650 ± 54	54 ± 11	18.1 ± 5.0	88	1610
PEIT-5	60.1 ± 2.1	1870 ± 70	32 ± 10	9.7 ± 3.6	93	2160
PEIT-10	68.7 ± 0.2	1860 ± 34	14 ±3	5.9 ± 1.3	99	2380
PEIT-20	67.6 ± 1.9	1940 ± 44	13 ± 6	6.1 ± 2.9	117	2250

a Toughness (*U*) was calculated from the integral area of stress–strain curves.

b Glass transition temperature (*T*_*g*_) was determined from the maximum of the tan δ curves by DMA.

c Storage modulus (*E’*) at 25 °C obtained from storage modulus curves by DMA.

PEIT polyesters synthesized here exhibit competitive mechanical performances compared with previously reported IS-based polyester-carbonates: a feature largely attributable to the crystallization behavior, which is partly retained even with IS incorporation. For instance, fully rigid IS-based polyester-carbonates (PCCITs) show tensile strengths from 43 to 58 MPa and modulus from 360 to 1400 MPa as IS content increases from 0 to 60%.^57^ Similarly, PEIT-10 (68.7 MPa) shows tensile strength slightly higher than what was reported for fully IS-based poly(isosorbide carbonate) (PIC) (67.6 MPa).^58^ This improvement is likely deduced from the ability of PEITs to crystallize more efficiently, resulting in a more rigid and structured polymer matrix, which further underscores an advantageous balance of rigidity and crystallinity. Theoretically, increasing rigid units should transition the polymer from ductile to brittle fracture, but here, the elongation at break and toughness initially increased before decreasing. Notably, PEIT-1 showed elongation at break and toughness approximately 2.4 and 2.3 times higher than that of PET, respectively. Scanning electron microscopy of the fracture surfaces (Figure 4c) revealed that the surface of PET was smoother with fewer cracks, while PEIT-1 and PEIT-5 had more, orderly, serrated cracks. This was especially notable in PEIT-1. These cracks can dissipate significant energy under stress, increasing elongation at the break and toughness. Thus, it is hypothesized that a trace of IS interacts with flexible chain segments, dispersing stress and enhancing toughness. A comparable phenomenon was observed for PET copolyesters with a small amount of furan units, where toughness increased 3.6 times with only 0.99 mol % furan content.^59^ Similarly, in poly(butylene succinate) (PBS) systems, incorporation of minimal amounts of comonomers, such as tartaric or citric acids, has been shown to significantly enhance the elongation at break and toughness, further highlighting how minor structural adjustments can significantly improve the mechanical performance.^60^

To further investigate the impact of IS units on the mechanical properties of PEIT copolyesters at different operating temperatures, we performed dynamic mechanical analysis. Figure 4d,e shows the changes in storage modulus and loss tangent of the materials at different temperatures. As shown in Figure 4d, the copolyesters containing IS exhibited significantly larger storage modulus compared to PET. At room temperature, the storage modulus increased from 1540 to 2380 MPa with increasing IS content, mirroring the trend observed for Young’s modulus of the copolyesters. This is again explained by the cyclic structure of IS units enhancing the rigidity of the polymer chains. However, the storage modulus of PEIT-20 at room temperature was not the highest among the synthesized copolyesters because of the greatly reduced crystallinity and lower molecular weight. Nevertheless, this amorphous material still exhibited higher rigidity than pure PET.

Examining the modulus at high temperatures revealed a more pronounced modulus enhancement for the copolyesters. For instance, when the temperature exceeded 70 °C, the storage modulus of PET significantly decreased, dropping below 10 MPa at 100 °C, whereas the storage modulus of PEIT-20 remained above 1200 MPa, which is approximately 149 times the storage modulus of PET. This indicates that PEIT is more suitable for high-temperature applications compared to traditional PET. Additionally, an increase in modulus was observed for PEIT-1, PEIT-5, and PEIT-10 when the temperature was above 140 °C, which could be attributed to cold crystallization during the heating process. The temperature at which tan δ reaches its maximum in Figure 4e represents the *T*_*g*_ of the material. It is evident that the addition of IS significantly increased the *T*_*g*_ values of the copolyesters, which is consistent with the DSC results.

### Optical Properties

**Figure 5:**
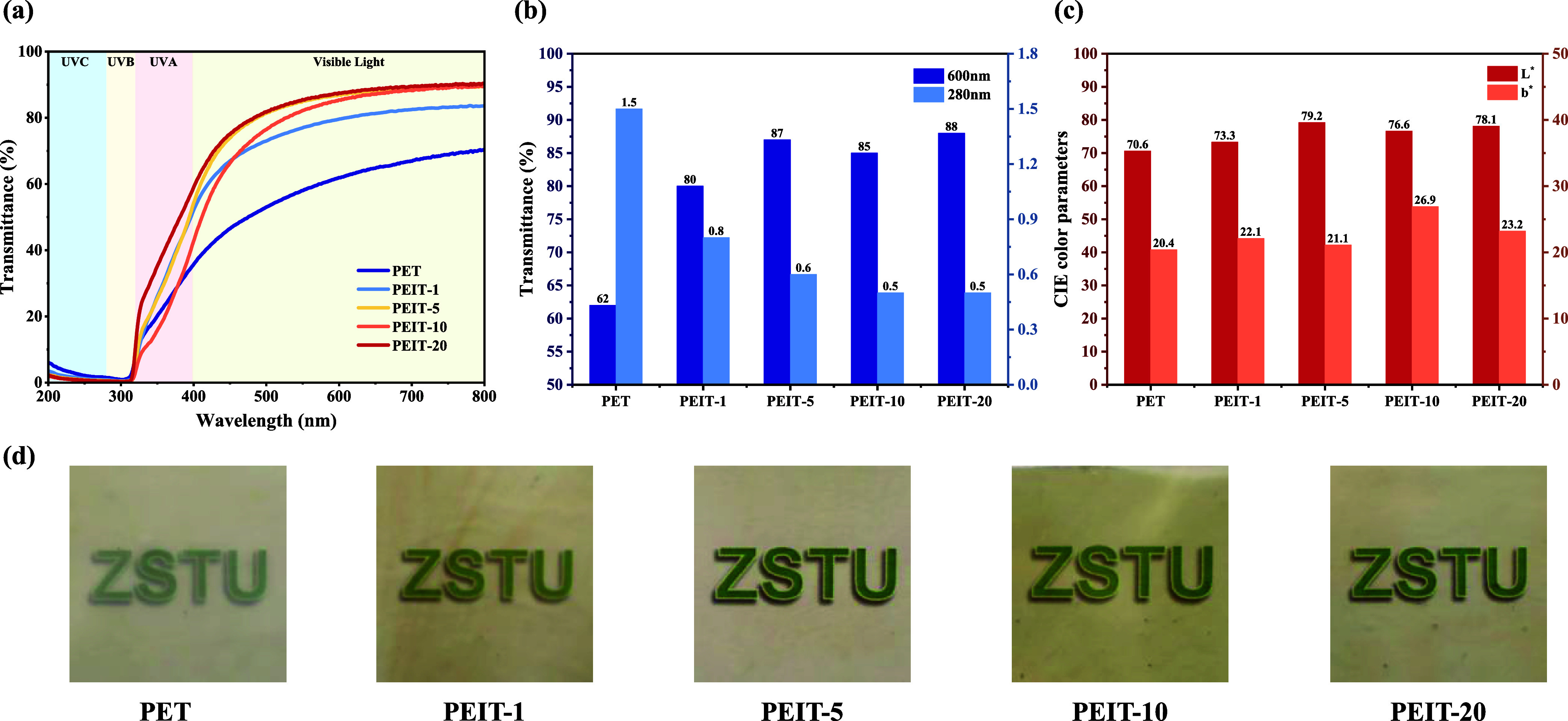
Optical properties of PEIT copolyesters with different isosorbide contents. a) UV–vis transmittance curves of melt-pressed copolyester films. b) Transmittance values at 600 and 280 nm. c) CIE color parameters of copolyester films. d) Images of PEIT films (0.2 mm thick).

Copolyester films with different IS contents and a thickness of 0.2 mm were also tested for their transmittance using UV–vis spectroscopy. Figure 5a shows the transmittance of all PEIT copolyester films in the wavelength range of 200–800 nm, reflecting the films’ transparency and UV shielding capabilities. The highest transmittance of the PEIT copolyester films reached 90%.

We further investigated the optical properties at wavelengths of 600 and 280 nm, representing the visible and UV regions, respectively (Figure 5b). At 600 nm, even with the addition of a small amount (1 mol %) of IS, the transmittance of the film significantly increased from 62 to 80%. This is likely due to the unique chiral structure of IS, which inhibits crystallization while aiding in the reflection and refraction of light, thereby greatly enhancing the film’s transparency. As the IS content increased, the transmittance continued to rise, with PEIT-5, PEIT-10, and PEIT-20 showing transmittance above 85% at 600 nm. However, the transmittance of PEIT-10 slightly decreased, possibly due to the nonuniformity in the amorphous region caused by higher IS content, leading to uneven light scattering and refraction. When the IS content reached 20%, the copolyester became completely amorphous, alleviating this phenomenon. At 280 nm, the transmittance of the film slightly decreased from 1.5 to 0.8% with the increase in IS content.

Subsequently, the CIE color parameters of the copolyester films were measured, and the L* and b* values for the films are presented in Figure 5c. The L* value represents the brightness of the films, with higher values indicating greater transparency and reduced light scattering. The b* value reflects the yellow-blue chromaticity, where a higher b* corresponds to a more pronounced yellow hue. The L* initially increases with IS content, peaking at PEIT-5, which exhibits the highest brightness and transparency. However, at higher IS contents (e.g., PEIT-10 and PEIT-20), the L* slightly decreases, following the trend of the transmittance. In contrast, the b* gradually increases with IS content. This is primarily attributed to the thermal oxidation of IS during the polymerization process, generating chromophoric byproducts that cause yellowing.

Furthermore, these trends can also be visualized in digital photographs of film samples (Figure 5d). PET exhibited poor transparency, and it is difficult to see the image behind the film. The films became increasingly transparent with a higher IS content. Notably, the image behind the film was clearly visible to the naked eye when the IS content reached 5% and exhibited a low degree of yellowness. These findings confirm that the incorporation of IS enhances the optical properties of the copolyesters, emphasizing the great potential of PEIT as a transparent packaging material.

### Barrier Properties

For packaging films and many other products, gas barrier properties are very important. We investigated the nitrogen (N_2_), oxygen (O_2_), and water vapor barrier properties of PEIT copolyester films using a differential pressure gas permeation test. The permeability coefficients of different films, including N_2_, O_2_, and water vapor, are presented in Table 5. The results show that the N_2_ and O_2_ permeability coefficients of the films initially decreased and then increased when the IS content increased. Previous studies have shown that when the layered crystals in the polymer are aligned parallel to the gas diffusion direction, they effectively hinder gas diffusion, resulting in good gas barrier properties.^61^ Although the crystalline structure of the copolyester with a small amount of IS remains similar to that of PET, differences in internal crystal arrangement may contribute to the observed enhancement in barrier performance. Additionally, crystallinity plays a critical role in the gas permeability coefficient. As the IS content increased, the inhibition of crystallization became more pronounced, leading to a modest decrease in barrier performance for N_2_ and O_2_. Notably, the overall O_2_ barrier performance was slightly inferior to N_2_, which can be attributed to the smaller molecular size of O_2_ (346 pm) compared to N_2_ (364 pm),^62^ making the diffusion of O_2_ molecules through the polymer matrix easier (Table 5).

**Table 5:** Gas Permeability Coefficient of PEIT Copolyesters with Different Isosorbide Contents

Sample	N_2_^a^ (10^–16^·cm^3^·cm/cm^2^·s·Pa)	O_2_^b^ (10^–16^·cm^3^·cm/cm^2^·s·Pa)	Water vapor^c^(10^–16^·cm^3^·cm/cm^2^·s·Pa)
PET	5.1 (± 3.0)	7.0 (± 0.2)	25.0 (± 6.8)
PEIT-1	4.6 (± 0.3)	6.3 (± 0.5)	29.0 (± 7.7)
PEIT-5	5.7 (± 0.4)	7.3 (± 0.4)	59.0 (± 5.3)
PEIT-10	7.5 (± 0.4)	8.9 (± 0.1)	110.0 (± 21.0)
PEIT-20	9.3 (± 0.2)	9.8 (± 0.4)	390.0 (± 41.0)

a N_2_ permeability coefficient, at 23 °C, 50% RH.

b O_2_ permeability coefficient, at 23 °C, 50% RH.

c Water vapor transmission rate, at 38 °C, 90% RH.

In contrast, the water vapor permeability of the films showed a continuous upward trend with increasing IS content. Water vapor molecules, with smaller molecular dimensions (294–302 pm),^63^ diffuse more easily through the polymer matrix. Additionally, the polar ester groups in the copolyester structure can interact with water molecules, further increasing the water vapor permeability. At higher IS content (PEIT-10 and PEIT-20), the water vapor permeability coefficient showed a significant increase, likely due to the enhanced hydrophilicity of the films, which facilitates water molecule diffusion. In conclusion, although the incorporation of IS slightly reduces the gas barrier performance compared to PET, the permeability coefficients for N_2_, O_2_, and water vapor remain within an acceptable range for practical applications.

### Degradation Properties

To demonstrate that the presence of IS units can be utilized to tune and accelerate the hydrolytic degradation of PEITs, we conducted alkaline degradation experiments with the copolyester films in 0.1 M NaOH solution at 58 °C. As shown in Table 5, the results illustrate an increasing weight loss as a function of IS content. After 50 days, the residual mass of PEIT-20 was 78%, compared to 94% for PET, corresponding to approximately 3.5 times higher weight loss for PEIT-20. Simultaneously, the molecular weight of all copolyesters decreased to varying degrees (Table S1). The incorporation of IS facilitated the molecular weight reduction of copolyesters. For example, the *M*_*w*_ of PET decreased from 42,000 to 40,100 g/mol, while PEIT-1 decreased from 56,600 to 33,700 g/mol. These results confirm that the incorporation of even small amounts of IS units significantly enhances the degradability of the copolyesters. This conclusion is further supported by DSC and XRD analyses (Figures S7–S10). Throughout the degradation process, PET maintained a stable crystallization behavior. In contrast, PEIT-20 exhibited distinct changes. During hydrolysis, PEIT-20 initially formed unstable, low-crystalline regions, which were subsequently disrupted as chain scission progressed. This behavior can be explained by the preferred hydrolysis in the amorphous regions, as also evidenced by the initial weight loss.

**Figure 6:**
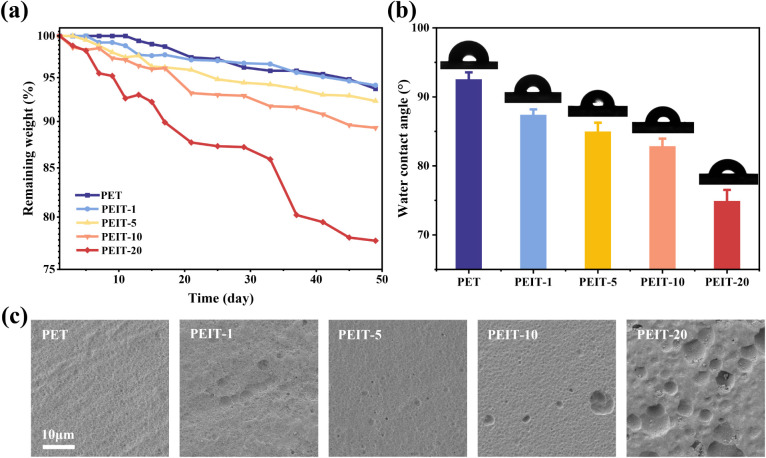
Degradation of PEIT copolyesters with different isosorbide contents. a) Weight loss of melt-pressed PEIT copolyester films during alkaline hydrolysis. b) Water contact angle of PEIT copolyesters. c) SEM micrographs of PEIT copolyesters after degradation in 0.1 M NaOH solution for 15 days.

The hydrophilicity of a copolyester is a key factor influencing the hydrolytic degradation process, as increased hydrophilicity facilitates water diffusion from the surface into the polymer matrix, enhancing the degradation rate.^64^ The hydrophilicity of PEIT copolyesters was investigated by measuring the water contact angle (WCA) of the film samples. Lower contact angle values indicate higher hydrophilicity. As shown in Figure 6, PET exhibited a clearly hydrophobic character with a WCA of 93°. With the addition of IS, the WCA of PEIT copolyesters gradually decreased to 75°, indicating the enhanced hydrophilicity of the polymer surface, which is expected to promote the hydrolytic degradation process. After 15 days of hydrolysis, we examined the surface morphology of different copolyester films using SEM (Figure 6). Films with higher IS content showed very rough surfaces, while those with low or no IS content still had relatively smooth surfaces. This further confirmed that the introduction of hydrophilic ether rings promotes hydrolytic degradation, leading to a more hydrophilic surface and facilitating overall material degradation by providing hydrolysis-sensitive linkages. This further leads to the release of oligomers that due to lower molecular weight are expected to be more susceptible to subsequent biodegradation.^65^ The potential of this approach is further supported by studies, where high molecular weight PET was shown to be nearly inert against enzymatic hydrolysis, while the enzymes showed high activity for pretreated low molecular weight PET.^66^

## Conclusions

A series of copolyesters with tunable thermal and mechanical properties and degradation rates were successfully fabricated by utilizing different amounts of biobased IS, as a rigid aliphatic building block. The copolyesters were synthesized from DMT, EG, and IS via a two-step reaction, i.e., transesterification and polycondensation. Five common catalysts were screened for the transesterification stage, and the highest IS and EG conversion rates in both biphasic (DMT/IS) or triphasic (DMT/IS/EG) systems were obtained with TBT as a catalyst. Benefiting from the efficiency of TBT, the compositions of PEITs were close to the feed ratios, and the resulting copolyesters possessed high molecular weights (*M*_*n*_ = 18,000 – 38,900 g/mol, *M*_*w*_ = 34,400 – 56,600 g/mol) and intrinsic viscosities (0.64–0.86 dL/g). By varying the IS content from 0 to 20 mol %, the *T*_*g*_ significantly increased from 80 to 101 °C, the tensile strength from 58.8 to 68.7 MPa, and Young’s modulus from 1.7 to 1.9 GPa. Notably, PEIT-1 showed an elongation at break approximately 2.4 times that of PET and an increase in toughness by about 2.3 times. This is explained by the trace of IS (1 mol %) promoting the transesterification reaction, which subsequently increased the molecular weight. Additionally, the cracks observed in the fracture surface are in an orderly serrated pattern, dissipating most of the capacity under stress. Meanwhile, the ether bond of IS increased the hydrophilicity of the PEIT films, reducing the water contact angle on the film surface from 93° to 75°. Consequently, the weight loss of PEIT-20 was approximately 3.5 times higher than what was observed for PET under alkaline conditions. This preliminary degradation study shows that the incorporation of IS units increases the hydrophilicity of the material, which promotes hydrolytic degradation by facilitating water penetration into the polymer matrix. In summary, this work presents a method for synthesizing high-performance copolyesters with increased susceptibility to hydrolytic degradation using alicyclic monomers. These copolyesters have great potential in high-performance and high-temperature applications, such as thermal packaging materials.

## Supporting Information Available

FTIR spectra of PEITs, ^1^H NMR spectra of PEITs, APC curves of PEITs, frequency sweep measurement of PEIT transesterification products, DSC curves of transesterification products and final product of PEIT-1, molecular weight of PEITs before and after degradation, the first heating DSC curves of PET and PEIT-20 after degradation in 0.1 M NaOH solution for 0, 1, and 50 days, the wide-angle X-ray diffraction patterns of PET and PEIT-20 after degradation in 0.1 M NaOH solution for 0 and 50 days (PDF)

## References

[ref1] Pan D.; Su F.; Liu C.; Guo Z. Research progress for plastic waste management and manufacture of value-added products. Adv. Compos. Hybrid Mater. 2020, 3 (4), 443–461. 10.1007/s42114-020-00190-0.

[ref2] Nanda S.; Patra B. R.; Patel R.; Bakos J.; Dalai A. K. Innovations in applications and prospects of bioplastics and biopolymers: A review. Environ. Chem. Lett. 2022, 20 (1), 379–395. 10.1007/s10311-021-01334-4. 34867134 PMC8629338

[ref3] Walker S.; Rothman R. Life cycle assessment of bio-based and fossil-based plastic: A review. J. Cleaner Prod. 2020, 261, 121158 10.1016/j.jclepro.2020.121158.

[ref4] Ali S. S.; Elsamahy T.; Koutra E.; Kornaros M.; El-Sheekh M.; Abdelkarim E. A.; Zhu D.; Sun J. Degradation of conventional plastic wastes in the environment: A review on current status of knowledge and future perspectives of disposal. Sci. Total Environ. 2021, 771, 144719 10.1016/j.scitotenv.2020.144719. 33548729

[ref5] Chamas A.; Moon H.; Zheng J.; Qiu Y.; Tabassum T.; Jang J. H.; Abu-Omar M.; Scott S. L.; Suh S. Degradation Rates of Plastics in the Environment. ACS Sustainable Chem. Eng. 2020, 8 (9), 3494–3511. 10.1021/acssuschemeng.9b06635.

[ref6] Nicholson S. R.; Rorrer N. A.; Carpenter A. C.; Beckham G. T. Manufacturing energy and greenhouse gas emissions associated with plastics consumption. Joule 2021, 5 (3), 673–686. 10.1016/j.joule.2020.12.027.

[ref7] Stegmann P.; Daioglou V.; Londo M.; van Vuuren D. P.; Junginger M. Plastic futures and their CO(2) emissions. Nature 2022, 612 (7939), 272–276. 10.1038/s41586-022-05422-5. 36477132

[ref8] Zheng J.; Suh S. Strategies to reduce the global carbon footprint of plastics. Nat. Clim. Change 2019, 9 (5), 374–378. 10.1038/s41558-019-0459-z.

[ref9] Stubbins A.; Law K. L.; Muñoz S. E.; Bianchi T. S.; Zhu L. Plastics in the Earth system. Science 2021, 373 (6550), 51–55. 10.1126/science.abb0354. 34210876

[ref10] Lau W. W. Y.; Shiran Y.; Bailey R. M.; Cook E.; Stuchtey M. R.; Koskella J.; Velis C. A.; Godfrey L.; Boucher J.; Murphy M. B.; et al. Evaluating scenarios toward zero plastic pollution. Science 2020, 369 (6510), 1455–1461. 10.1126/science.aba9475. 32703909

[ref11] MacLeod M.; Arp H. P. H.; Tekman M. B.; Jahnke A. The global threat from plastic pollution. Science 2021, 373 (6550), 61–65. 10.1126/science.abg5433. 34210878

[ref12] Woodard L. N.; Grunlan M. A. Hydrolytic Degradation and Erosion of Polyester Biomaterials. ACS Macro Lett. 2018, 7 (8), 976–982. 10.1021/acsmacrolett.8b00424. 30705783 PMC6350899

[ref13] Wang J.; Gao X.; Boarino A.; Célerse F.; Corminboeuf C.; Klok H.-A. Mechanical Acceleration of Ester Bond Hydrolysis in Polymers. Macromolecules 2022, 55 (22), 10145–10152. 10.1021/acs.macromol.2c01789.

[ref14] RameshKumar S.; Shaiju P.; O’Connor K. E.; P R. B. Bio-based and biodegradable polymers - State-of-the-art, challenges and emerging trends. Curr. Opin. Green Sustainable Chem. 2020, 21, 75–81. 10.1016/j.cogsc.2019.12.005.

[ref15] Elvers D.; Song C. H.; Steinbüchel A.; Leker J. Technology Trends in Biodegradable Polymers: Evidence from Patent Analysis. Polym. Rev. 2016, 56 (4), 584–606. 10.1080/15583724.2015.1125918.

[ref16] Kijchavengkul T.; Auras R.; Rubino M.; Selke S.; Ngouajio M.; Fernandez R. T. Biodegradation and hydrolysis rate of aliphatic aromatic polyester. Polym. Degrad. Stab. 2010, 95 (12), 2641–2647. 10.1016/j.polymdegradstab.2010.07.018.

[ref17] Kijchavengkul T.; Auras R.; Rubino M.; Alvarado E.; Camacho Montero J. R.; Rosales J. M. Atmospheric and soil degradation of aliphatic–aromatic polyester films. Polym. Degrad. Stab. 2010, 95 (2), 99–107. 10.1016/j.polymdegradstab.2009.11.048.

[ref18] Wang Y.; Wu J.; Koning C.E.; Wang H. Short-process synthetic strategies of sustainable isohexide-based polyesters towards higher molecular weight and commercial applicability. Green Chem. 2022, 24 (22), 8637–8670. 10.1039/D2GC02608B.

[ref19] Fang W.; Zhang Z.; Yang Z.; Zhang Y.; Xu F.; Li C.; An H.; Song T.; Luo Y.; Zhang S. One-pot synthesis of bio-based polycarbonates from dimethyl carbonate and isosorbide under metal-free condition. Green Chem. 2020, 22 (14), 4550–4560. 10.1039/D0GC01440K.

[ref20] Qian W.; Ma X.; Liu L.; Deng L.; Su Q.; Bai R.; Zhang Z.; Gou H.; Dong L.; Cheng W.; Xu F. Efficient synthesis of bio-derived polycarbonates from dimethyl carbonate and isosorbide: Regulating exo-OH and endo-OH reactivity by ionic liquids. Green Chem. 2020, 22 (16), 5357–5368. 10.1039/D0GC01804J.

[ref21] Park S.-A.; Eom Y.; Jeon H.; Koo J. M.; Lee E. S.; Jegal J.; Hwang S. Y.; Oh D. X.; Park J. Preparation of synergistically reinforced transparent bio-polycarbonate nanocomposites with highly dispersed cellulose nanocrystals. Green Chem. 2019, 21 (19), 5212–5221. 10.1039/C9GC02253H.

[ref22] Besse V.; Auvergne R.; Carlotti S.; Boutevin G.; Otazaghine B.; Caillol S.; Pascault J.-P.; Boutevin B. Synthesis of isosorbide based polyurethanes: An isocyanate free method. React. Funct. Polym. 2013, 73 (3), 588–594. 10.1016/j.reactfunctpolym.2013.01.002.

[ref23] Blache H.; Méchin F.; Rousseau A.; Fleury É.; Pascault J.-P.; Alcouffe P.; Jacquel N.; Saint-Loup R. New bio-based thermoplastic polyurethane elastomers from isosorbide and rapeseed oil derivatives. Ind. Crops Prod. 2018, 121, 303–312. 10.1016/j.indcrop.2018.05.004.

[ref24] Oprea S.; Potolinca V.-O.; Oprea V. Synthesis and properties of new crosslinked polyurethane elastomers based on isosorbide. Eur. Polym. J. 2016, 83, 161–172. 10.1016/j.eurpolymj.2016.08.020.

[ref25] Ryu J. U.; Yu H. J.; Seong J.; Kim H.-J.; Park J.; Lee J. S. Isosorbide-based Poly(arylene ether) biopolymer membranes for gas separation. J. Membr. Sci. 2024, 706, 122928 10.1016/j.memsci.2024.122928.

[ref26] Yang H.-S.; Cho S.; Lee M.; Eom Y.; Chae H. G.; Park S.-A.; Jang M.; Oh D. X.; Hwang S. Y.; Park J. Preparation of sustainable fibers from isosorbide: Merits over bisphenol-A based polysulfone. Mater. Des. 2021, 198, 109284 10.1016/j.matdes.2020.109284.

[ref27] Jasinska L.; Villani M.; Wu J.; van Es D.; Klop E.; Rastogi S.; Koning C. E. Novel, Fully Biobased Semicrystalline Polyamides. Macromolecules 2011, 44 (9), 3458–3466. 10.1021/ma200256v.

[ref28] Sawada R.; Ando S. Colorless, Low Dielectric, and Optically Active Semialicyclic Polyimides Incorporating a Biobased Isosorbide Moiety in the Main Chain. Macromolecules 2022, 55 (15), 6787–6800. 10.1021/acs.macromol.2c01288.

[ref29] Jasinska-Walc L.; Dudenko D.; Rozanski A.; Thiyagarajan S.; Sowinski P.; van Es D.; Shu J.; Hansen M. R.; Koning C. E. Structure and Molecular Dynamics in Renewable Polyamides from Dideoxy–Diamino Isohexide. Macromolecules 2012, 45 (14), 5653–5666. 10.1021/ma301091a.

[ref30] Kim D.; Kim I.-C.; Kwon Y.-N.; Myung S. Novel bio-based polymer membranes fabricated from isosorbide-incorporated poly(arylene ether)s for water treatment. Eur. Polym. J. 2020, 136, 109931 10.1016/j.eurpolymj.2020.109931.

[ref31] Saxon D. J.; Nasiri M.; Mandal M.; Maduskar S.; Dauenhauer P. J.; Cramer C. J.; LaPointe A. M.; Reineke T. M. Architectural Control of Isosorbide-Based Polyethers via Ring-Opening Polymerization. J. Am. Chem. Soc. 2019, 141 (13), 5107–5111. 10.1021/jacs.9b00083. 30835460

[ref32] Legrand S.; Jacquel N.; Amedro H.; Saint-Loup R.; Colella M.; Pascault J.-P.; Fenouillot F.; Rousseau A. Isosorbide and Tricyclodecanedimethanol for the Synthesis of Amorphous and High Tg Partially Biobased Copolyesters. ACS Sustainable Chem. Eng. 2020, 8 (40), 15199–15208. 10.1021/acssuschemeng.0c04679.

[ref33] Chebbi Y.; Kasmi N.; Majdoub M.; Cerruti P.; Scarinzi G.; Malinconico M.; Dal Poggetto G.; Papageorgiou G. Z.; Bikiaris D. N. Synthesis, Characterization, and Biodegradability of Novel Fully Biobased Poly(decamethylene-co-isosorbide 2,5-furandicarboxylate) Copolyesters with Enhanced Mechanical Properties. ACS Sustainable Chem. Eng. 2019, 7 (5), 5501–5514. 10.1021/acssuschemeng.8b06796.

[ref34] Qi J.; Wu J.; Chen J.; Wang H. An investigation of the thermal and (bio)degradability of PBS copolyesters based on isosorbide. Polym. Degrad. Stab. 2019, 160, 229–241. 10.1016/j.polymdegradstab.2018.12.031.

[ref35] Wang W.; Wu F.; Lu H.; Li X.; Yang X.; Tu Y. A Cascade Polymerization Method for the Property Modification of Poly(butylene terephthalate) by the Incorporation of Isosorbide. ACS Appl. Polym. Mater. 2019, 1 (9), 2313–2321. 10.1021/acsapm.9b00332.

[ref36] Jang H.; Park G.; Kwon S.; Park S.-I. Biodegradability of renewable isosorbide and sebacate-based copolyesters. Polym. Degrad. Stab. 2024, 228, 110931 10.1016/j.polymdegradstab.2024.110931.

[ref37] Zhang X.; Chen Y.; Ye M.; Wu J.; Wang H. Biodegradable Copolyesters with Unexpected Highly Blocky Microstructures and Enhanced Thermal Properties. ACS Sustainable Chem. Eng. 2022, 10 (14), 4438–4450. 10.1021/acssuschemeng.1c07993.

[ref38] Wilbon P. A.; Swartz J. L.; Meltzer N. R.; Brutman J. P.; Hillmyer M. A.; Wissinger J. E. Degradable Thermosets Derived from an Isosorbide/Succinic Anhydride Monomer and Glycerol. ACS Sustainable Chem. Eng. 2017, 5 (10), 9185–9190. 10.1021/acssuschemeng.7b02096.

[ref39] Qu D.; Zhang F.; Gao H.; Wang Q.; Bai Y.; Liu H. Studies on Isosorbide-enhanced Biodegradable Poly(ethylene succinate). Chem. Res. Chin. Univ. 2019, 35 (2), 345–352. 10.1007/s40242-019-8227-1.

[ref40] Wu J.; Qi J.; Lin Y.; Chen Y.; Zhang X.; Wu R.; Wang H. Lipase-Catalyzed Fully Aliphatic Copolyesters Based on Renewable Isohexide Isomers. ACS Sustainable Chem. Eng. 2021, 9 (4), 1599–1612. 10.1021/acssuschemeng.0c06733.

[ref41] Qu D.; Sun S.; Gao H.; Bai Y.; Tang Y. Biodegradable copolyester poly(butylene-co-isosorbide succinate) as hot-melt adhesives. RSC Adv. 2019, 9 (20), 11476–11483. 10.1039/C9RA01780A. 35520238 PMC9063263

[ref42] Chen J.; Wu J.; Qi J.; Wang H. Systematic Study of Thermal and (Bio)Degradable Properties of Semiaromatic Copolyesters Based on Naturally Occurring Isosorbide. ACS Sustainable Chem. Eng. 2019, 7 (1), 1061–1071. 10.1021/acssuschemeng.8b04717.

[ref43] Li X.-G.; Song G.; Huang M.-R.; Ohara T.; Yamada H.; Umeyama T.; Higashino T.; Imahori H. Cleaner synthesis and systematical characterization of sustainable poly(isosorbide-co-ethylene terephthalate) by environ-benign and highly active catalysts. J. Cleaner Prod. 2019, 206, 483–497. 10.1016/j.jclepro.2018.09.046.

[ref44] Saxon D. J.; Luke A. M.; Sajjad H.; Tolman W. B.; Reineke T. M. Next-generation polymers: Isosorbide as a renewable alternative. Prog. Polym. Sci. 2020, 101, 101196 10.1016/j.progpolymsci.2019.101196.

[ref45] Weinland D. H.; van Putten R.-J.; Gruter G.-J. M. Evaluating the commercial application potential of polyesters with 1,4: 3,6-dianhydrohexitols (isosorbide, isomannide and isoidide) by reviewing the synthetic challenges in step growth polymerization. Eur. Polym. J. 2022, 164, 110964 10.1016/j.eurpolymj.2021.110964.

[ref46] Weinland D. H.; van der Maas K.; Wang Y.; Bottega Pergher B.; van Putten R. J.; Wang B.; Gruter G. M. Overcoming the low reactivity of biobased, secondary diols in polyester synthesis. Nat. Commun. 2022, 13 (1), 7370 10.1038/s41467-022-34840-2. 36450717 PMC9712608

[ref47] Stanley N.; Chenal T.; Jacquel N.; Saint-Loup R.; Prates Ramalho J. P.; Zinck P. Organocatalysts for the Synthesis of Poly(ethylene terephthalate-co-isosorbide terephthalate): A Combined Experimental and DFT Study. Macromol. Mater. Eng. 2019, 304 (9), 1900298 10.1002/mame.201900298.

[ref48] Xie S.; Qian S.; Zhu K.; Sun L.; Chen W.; Chen S. Comparison of Eco-friendly Ti–M Bimetallic Coordination Catalysts and Commercial Monometallic Sb- or Ti-Based Catalysts for the Synthesis of Poly(ethylene-co-isosorbide terephthalate). ACS Omega 2023, 8 (22), 19237–19248. 10.1021/acsomega.2c07831. 37305258 PMC10249036

[ref49] Stanley N.; Chenal T.; Delaunay T.; Saint-Loup R.; Jacquel N.; Zinck P. Bimetallic Catalytic Systems Based on Sb, Ge and Ti for the Synthesis of Poly(ethylene terephthalate-co-isosorbide terephthalate). Polymers 2017, 9 (11), 590 10.3390/polym9110590. 30965895 PMC6418978

[ref50] Bersot J. C.; Jacquel N.; Saint-Loup R.; Fuertes P.; Rousseau A.; Pascault J. P.; Spitz R.; Fenouillot F.; Monteil V. Efficiency Increase of Poly(ethylene terephthalate-co-isosorbide terephthalate) Synthesis using Bimetallic Catalytic Systems. Macromol. Chem. Phys. 2011, 212 (19), 2114–2120. 10.1002/macp.201100146.

[ref51] Yoon W. J.; Hwang S. Y.; Koo J. M.; Lee Y. J.; Lee S. U.; Im S. S. Synthesis and Characteristics of a Biobased High-Tg Terpolyester of Isosorbide, Ethylene Glycol, and 1,4-Cyclohexane Dimethanol: Effect of Ethylene Glycol as a Chain Linker on Polymerization. Macromolecules 2013, 46 (18), 7219–7231. 10.1021/ma4015092.

[ref52] Abbasi M.; Kotek R. Effects of drawing process on crimp formation-ability of side-by-side bicomponent filament yarns produced from recycled, fiber-grade and bottle-grade PET. J. Text. Inst. 2019, 110 (10), 1439–1444. 10.1080/00405000.2019.1611523.

[ref53] Gandini A.; Lacerda T.M. Furan Polymers: State of the Art and Perspectives. Macromol. Mater. Eng. 2022, 307 (6), 2100902 10.1002/mame.202100902.

[ref54] Sahu P.; Thorbole A.; Gupta R. K. Polyesters Using Bioderived Furandicarboxylic Acid: Recent Advancement and Challenges toward Green PET. ACS Sustainable Chem. Eng. 2024, 12 (18), 6811–6826. 10.1021/acssuschemeng.4c01123.

[ref55] Irshad F.; Khan N.; Howari H.; Fatima M.; Farooq A.; Awais M.; Ayyoob M.; Tusief M. Q.; Virk R.; Hussain F. Recent Advances in the Development of 1,4-Cyclohexanedimethanol (CHDM) and Cyclic-Monomer-Based Advanced Amorphous and Semi-Crystalline Polyesters for Smart Film Applications. Materials 2024, 17 (18), 4568 10.3390/ma17184568. 39336309 PMC11432963

[ref56] Sánchez-Arrieta N.; de Ilarduya A. M.; Alla A.; Muñoz-Guerra S. Poly(ethylene terephthalate) copolymers containing 1,4-cyclohexane dicarboxylate units. Eur. Polym. J. 2005, 41 (7), 1493–1501. 10.1016/j.eurpolymj.2005.02.004.

[ref57] Zeng C.; Ren J.; Shen W.; Zhang S.; Ji P.; Wang C.; Wang H. Synthesis of Thermal-Resistant Polyester-Polycarbonate with Fully Rigid Structure from Biobased Isosorbide. Macromolecules 2024, 57 (13), 6284–6294. 10.1021/acs.macromol.4c00647.

[ref58] Wang Y.; Xie R.; Xu J.; Li Z.; Guo M.; Yang F.; Wu J.; Wang H. Strong, Tough, and Heat-Resistant Isohexide-Based Copolycarbonates: An Ecologically Safe Alternative for Bisphenol-A Polycarbonate. ACS Sustainable Chem. Eng. 2024, 12 (19), 7553–7565. 10.1021/acssuschemeng.4c01448.

[ref59] Zhang P.; Chen L.; Guo Y.; Zhang L.; Wang X.-L.; Wang Y.-Z. Hetero-Furan Unit” Drives a Spontaneous Leaf-Vein Bioinspired Multiscale Design for Ultra-Robust, Lightweight, and Recyclable Polymers. Macromolecules 2023, 56 (21), 8823–8833. 10.1021/acs.macromol.3c01054.

[ref60] Kim H.; Shin G.; Jang M.; Nilsson F.; Hakkarainen M.; Kim H. J.; Hwang S. Y.; Lee J.; Park S. B.; Park J.; Oh D. X.; Jeon H.; Koo J. M. Toward Sustaining Bioplastics: Add a Pinch of Seasoning. ACS Sustainable Chem. Eng. 2023, 11 (5), 1846–1856. 10.1021/acssuschemeng.2c06247.

[ref61] Bai H.; Huang C.; Xiu H.; Zhang Q.; Deng H.; Wang K.; Chen F.; Fu Q. Significantly Improving Oxygen Barrier Properties of Polylactide via Constructing Parallel-Aligned Shish-Kebab-Like Crystals with Well-Interlocked Boundaries. Biomacromolecules 2014, 15 (4), 1507–1514. 10.1021/bm500167u. 24617940

[ref62] Park D.; Ju Y.; Kim J.-H.; Ahn H.; Lee C.-H. Equilibrium and kinetics of nitrous oxide, oxygen and nitrogen adsorption on activated carbon and carbon molecular sieve. Sep. Purif. Technol. 2019, 223, 63–80. 10.1016/j.seppur.2019.04.051.

[ref63] Malomuzh N. P.; Zhyganiuk I. V.; Timofeev M. V. Nature of H-bonds in water vapor. J. Mol. Liq. 2017, 242, 175–180. 10.1016/j.molliq.2017.06.127.

[ref64] Höglund A.; Odelius K.; Hakkarainen M.; Albertsson A.-C. Controllable Degradation Product Migration from Cross-Linked Biomedical Polyester-Ethers through Predetermined Alterations in Copolymer Composition. Biomacromolecules 2007, 8 (6), 2025–2032. 10.1021/bm070292x. 17521165

[ref65] Aarsen C. V.; Liguori A.; Mattsson R.; Sipponen M. H.; Hakkarainen M. Designed to Degrade: Tailoring Polyesters for Circularity. Chem. Rev. 2024, 124 (13), 8473–8515. 10.1021/acs.chemrev.4c00032. 38936815 PMC11240263

[ref66] Guo B.; Lopez-Lorenzo X.; Fang Y.; Bäckström E.; Capezza A. J.; Vanga S. R.; Furó I.; Hakkarainen M.; Syrén P. O. Fast Depolymerization of PET Bottle Mediated by Microwave Pre-Treatment and An Engineered PETase**. ChemSuschem 2023, 16 (18), e202300742 10.1002/cssc.202300742. 37384425

