# Supplementary material for: Designing Biobased Poly(ethylene-co-isosorbide terephthalate) Copolyesters with Tunable Properties and Degradability
Source: Biomacromolecules. 2025 Mar 8;26(4):2304–16. doi: 10.1021/acs.biomac.4c01630 (PMC12004518; doi:10.1021/acs.biomac.4c01630)
Supplement: Supplementary file 1 — bm4c01630_si_001.pdf [file bm4c01630_si_001.pdf]

Designing Bio-based Poly(ethylene isosorbide terephthalate) (PEIT) with Tunable Properties and Degradability

Dan Li,[a b] Youbing Li, [a b] Yu Zhang, [a] Yunsheng Xu, [a b*] Xianming Zhang, [a b] and Minna Hakkarainen [c*]

[a] School of Materials Science and Engineering, Zhejiang Sci-Tech University, Hangzhou 310018, China

[b] Zhejiang Provincial Innovation Center Advanced Textile Technology, Shaoxing 312030, China

[c] Department of Fibre and Polymer Technology, KTH Royal Institute of Technology, Teknikringen 58, 100 44 Stockholm, Sweden

Correspondence: Yunsheng Xu, Email: xuyunsheng1019@zstu.edu.cn

Minna Hakkarainen, Email: minna@kth.se

This Supporting Information contains 1 table and 10 figures in 12 pages.

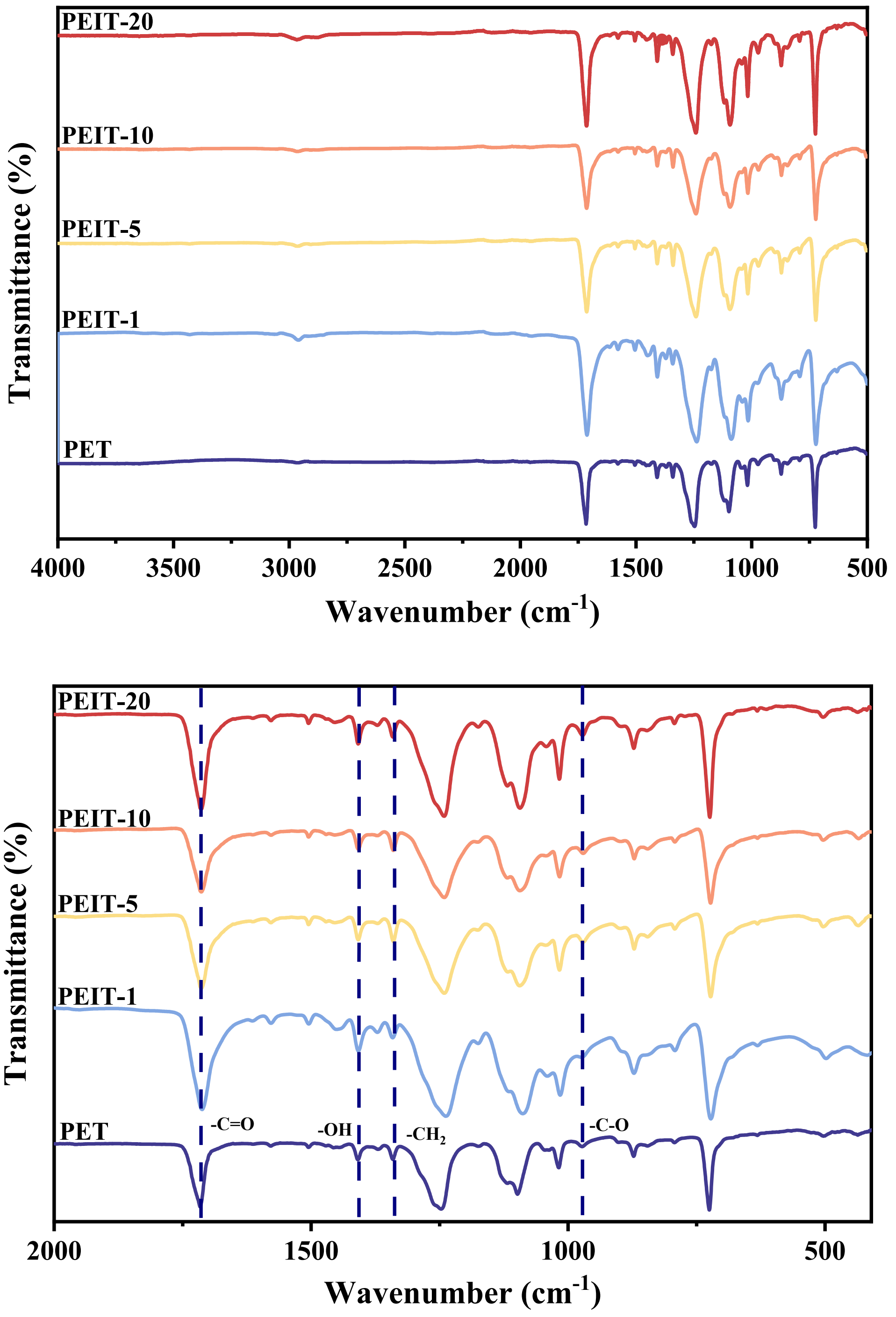

Figure S1. The FTIR spectra of PEIT copolyesters with different isosorbide contents.

(a)

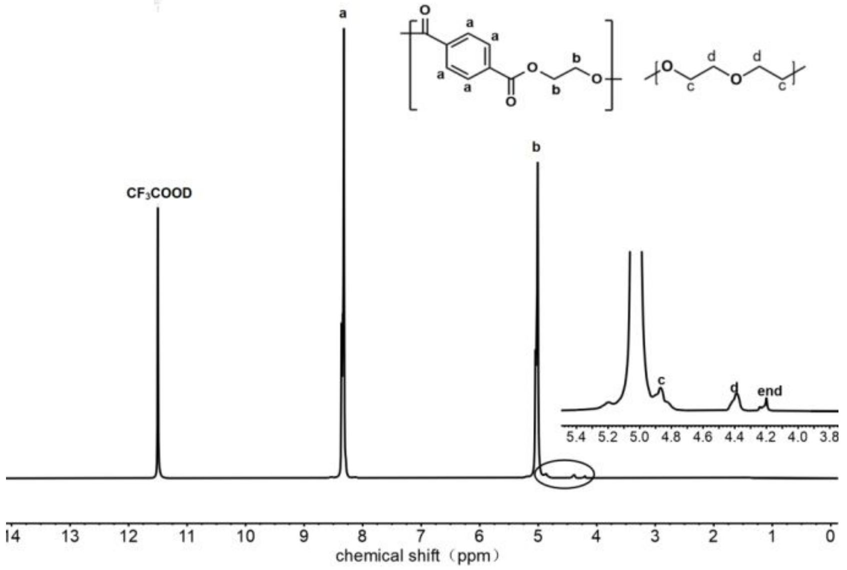
CF3COOD
a
b
c
d
end
5.4 5.2 5.0 4.8 4.6 4.4 4.2 4.0 3.8
14 13 12 11 10 9 8 7 6 5 4 3 2 1 0
chemical shift（ppm）

(b)

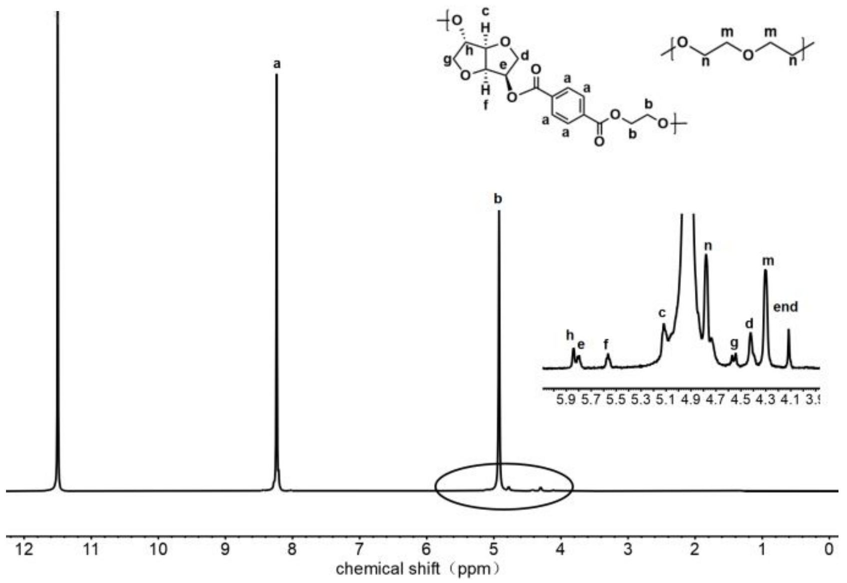
a
b
c
d
e
f
g
h
m
n
end
5.9 5.7 5.5 5.3 5.1 4.9 4.7 4.5 4.3 4.1
12 11 10 9 8 7 6 5 4 3 2 1 0
chemical shift（ppm）

(c)

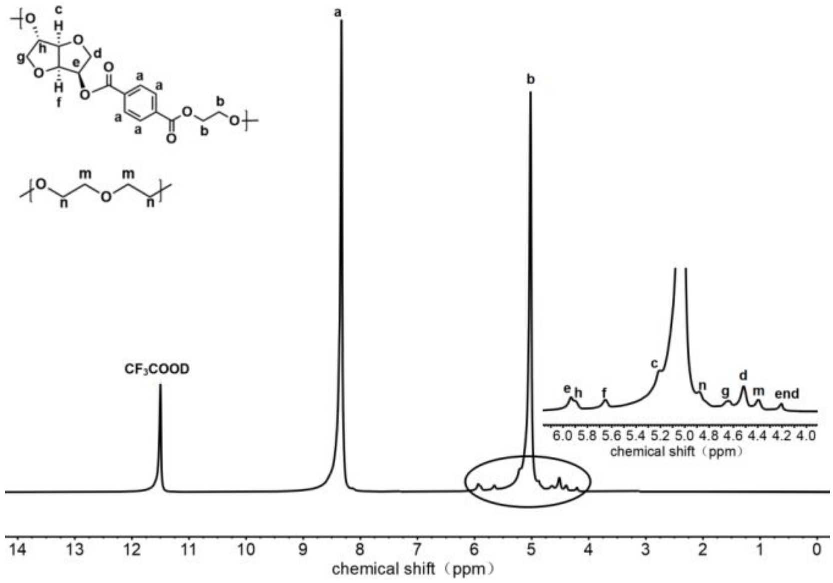
CF₃COOD
a
b
e h f c n d g m end
6.0 5.8 5.6 5.4 5.2 5.0 4.8 4.6 4.4 4.2 4.0
chemical shift（ppm）
14 13 12 11 10 9 8 7 6 5 4 3 2 1 0
chemical shift（ppm）

(d)

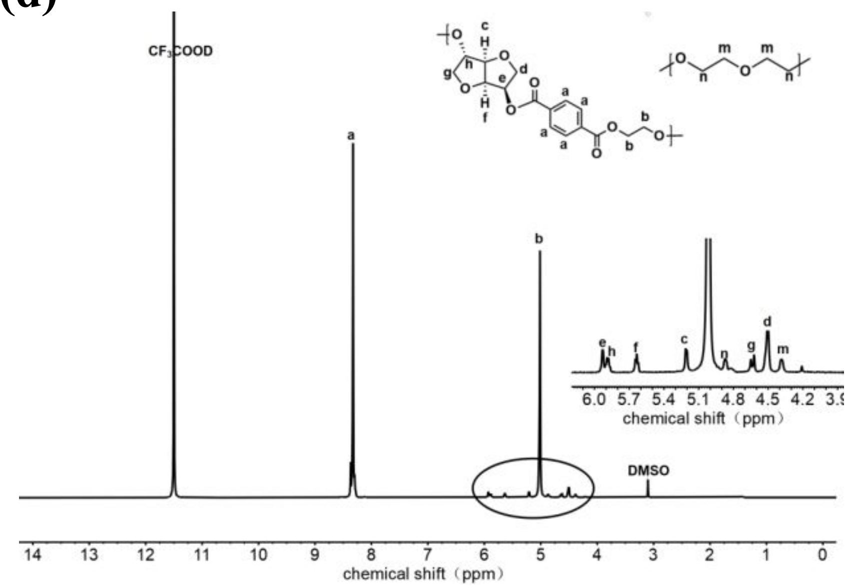
CF₃COOD
a
b
e h f c n g d m
6.0 5.7 5.4 5.1 4.8 4.5 4.2
chemical shift（ppm）
DMSO
14 13 12 11 10 9 8 7 6 5 4 3 2 1 0
chemical shift（ppm）

(e)

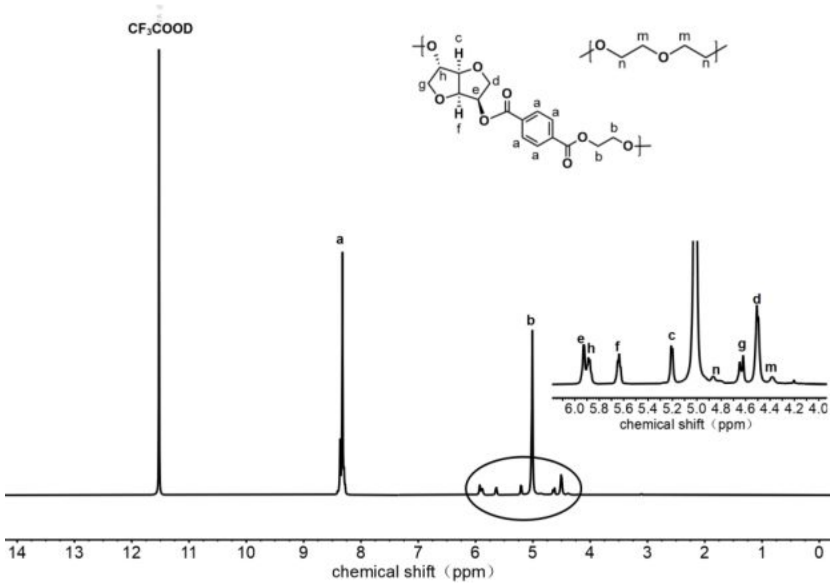

Figure S2. ^{1}H-NMR spectra of PEIT copolyesters with different isosorbide contents recorded in a mixed solvent of DMSO/CF3COOD at 400 MHz. (a) PET; (b) PEIT-1; (c) PEIT-5; (d) PEIT-10; (e) PEIT-20.

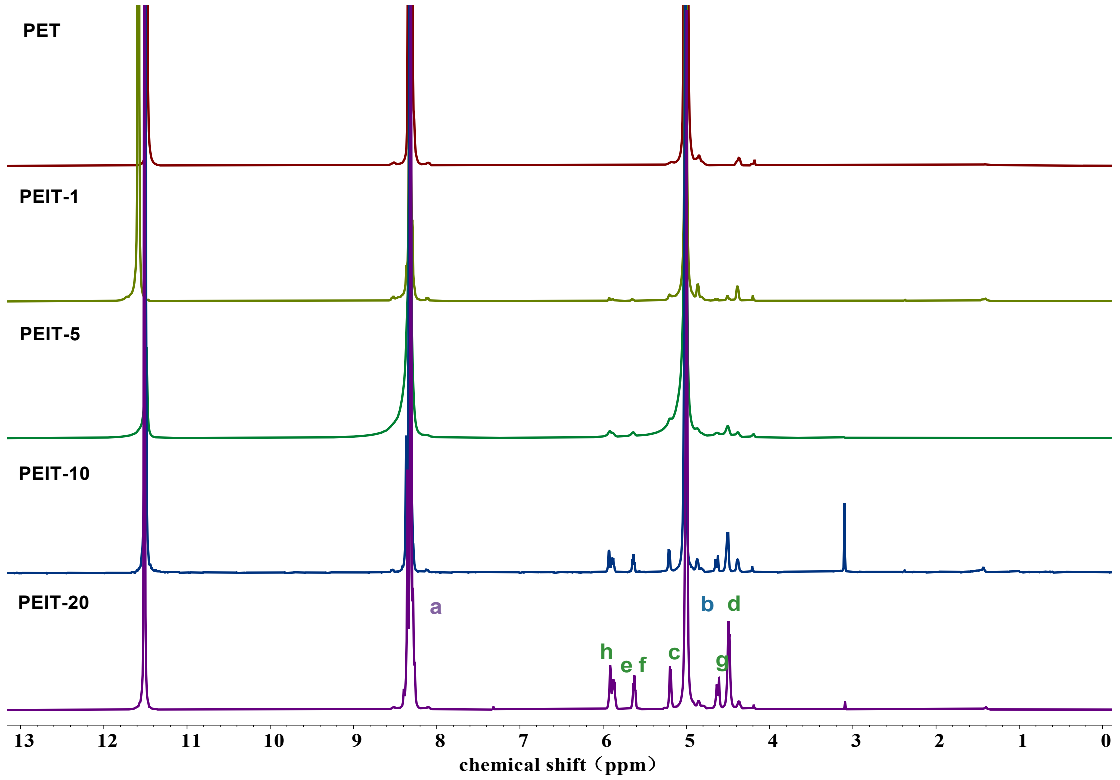

Figure S3. Full-range ^{1}H-NMR spectra of PEIT copolyesters with different isosorbide contents recorded in a mixed solvent of DMSO/CF3COOD at 400 MHz.

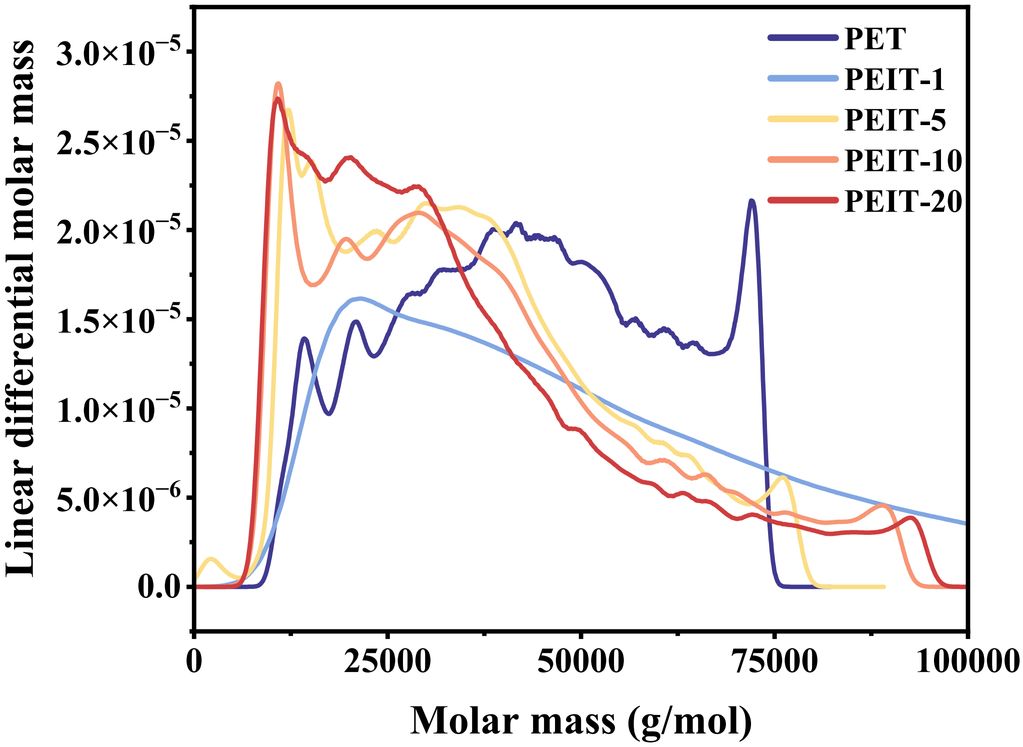

Figure S4. APC curves of PEIT copolyesters with different isosorbide contents.

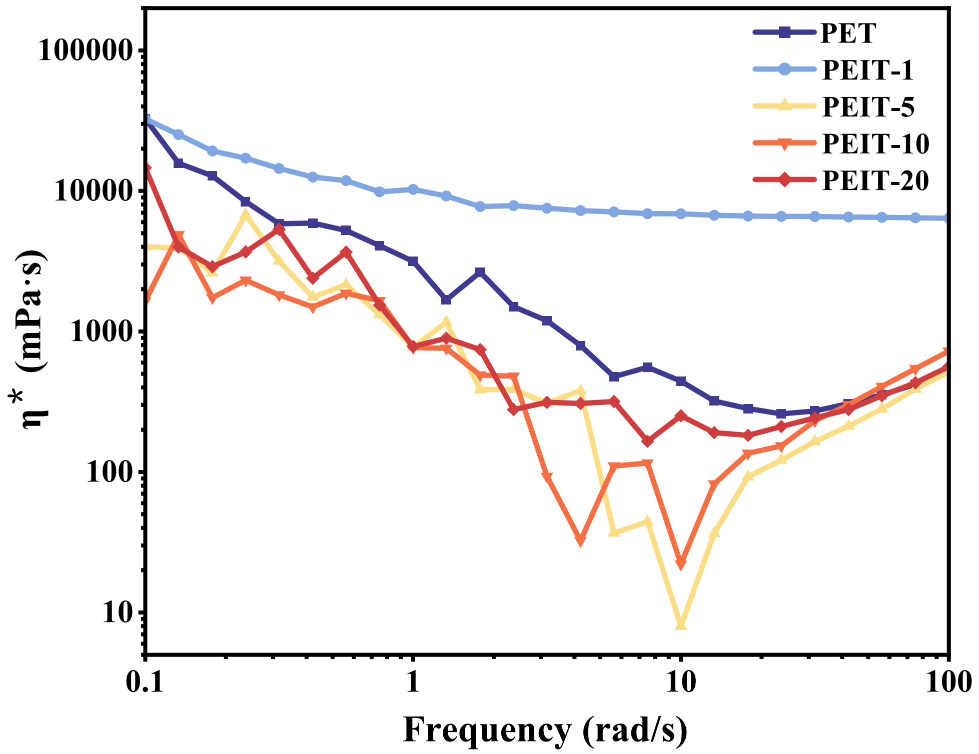

Figure S5. Frequency sweep measurements at 265 ℃ of PEIT oligomers with different isosorbide contents.

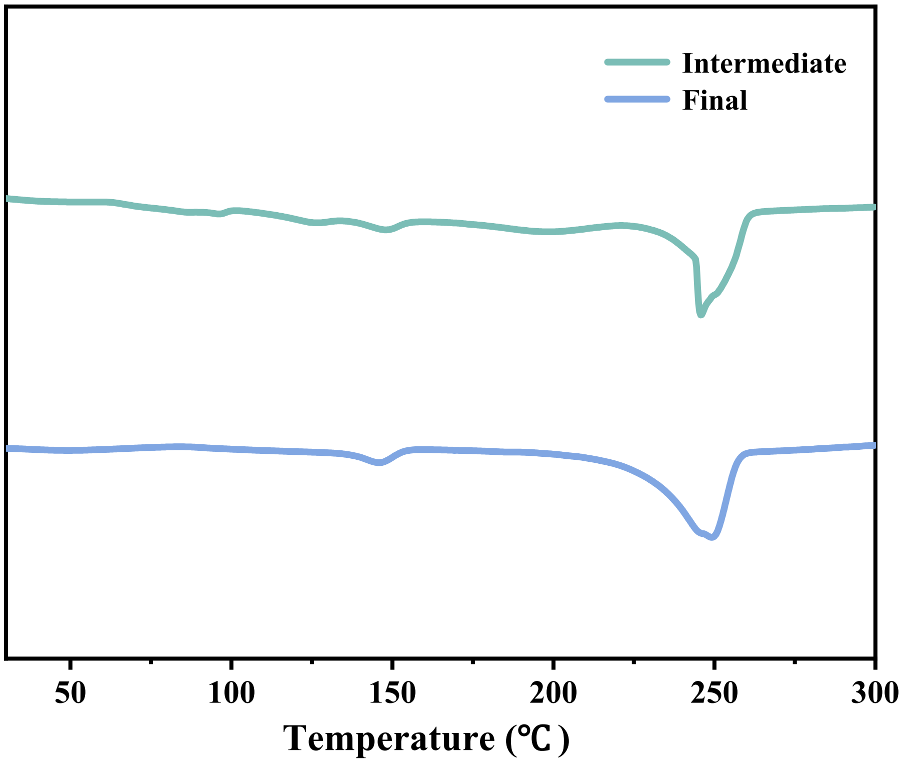

Figure S6. The DSC curves of the intermediate product and final product of PEIT-1.

Table S1. Molecular Weight of PEIT Copolyesters Before and After Degradation.

Sample	Before degradation			After degradation		
	M_n[c] (g/mol)	M_w[c] (g/mol)	$Đ$[c] (M_w/M_n)	M_n[c] (g/mol)	M_w[c] (g/mol)	$Đ$[c] (M_w/M_n)
**PET**	28800	42000	1.5	26700	40100	1.5
**PEIT-1**	38900	56600	1.5	21500	33700	1.6
**PEIT-5**	22200	35700	1.6	17400	32400	1.9
**PEIT-10**	19600	37100	1.9	16100	29100	1.8
**PEIT-20**	18000	34400	1.9	15600	27600	1.8

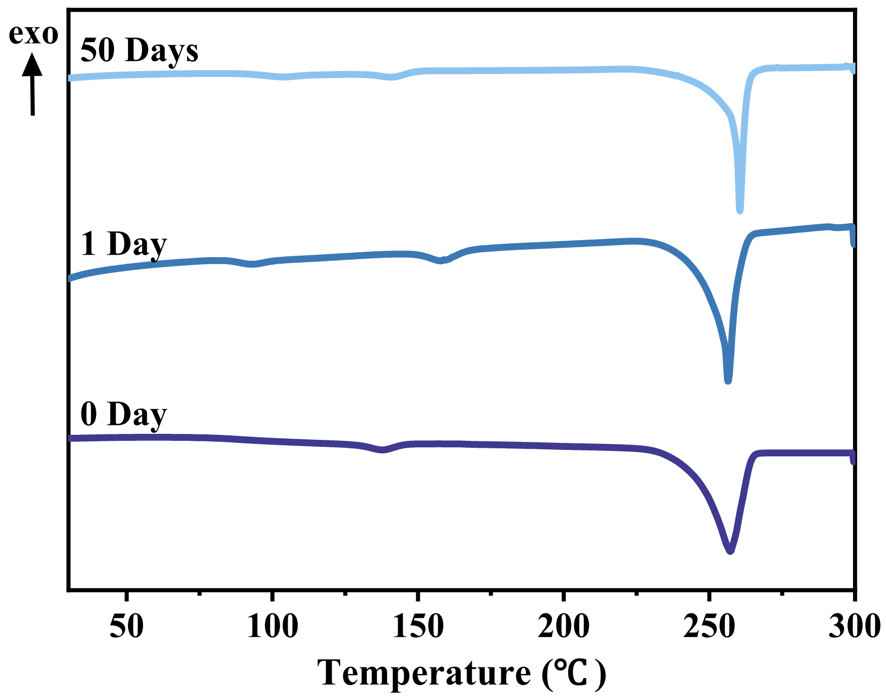

Figure S7. The first heating DSC curves of PET after degradation in 0.1M NaOH solution for 0, 1 and 50 days

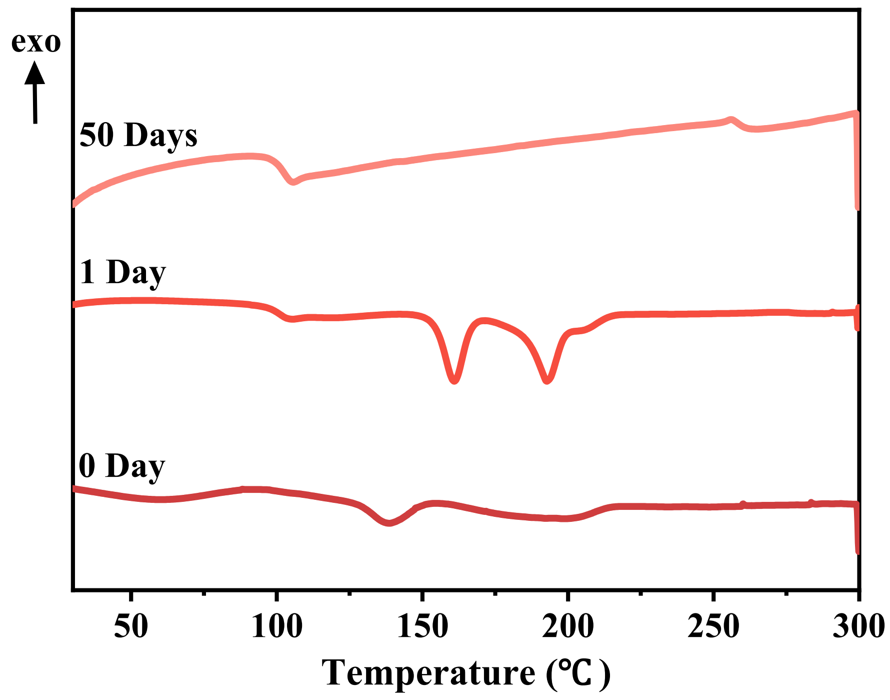

Figure S8. The first heating DSC curves of PEIT-20 after degradation in 0.1M NaOH solution for 0, 1 and 50 days.

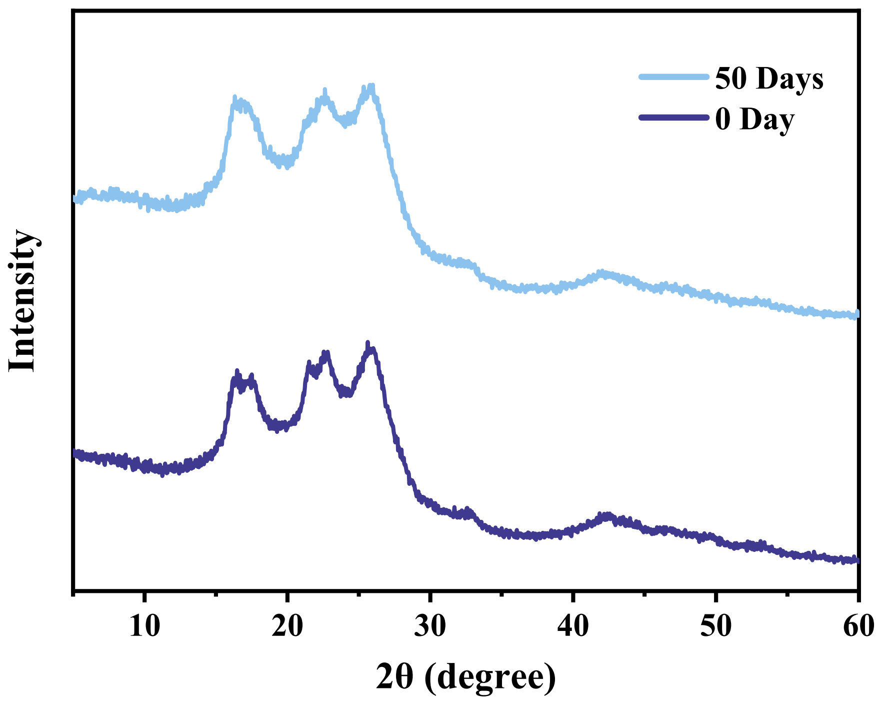

Figure S9. The wide angle X-ray diffraction patterns of PET after degradation in 0.1M NaOH solution for 0 and 50 days

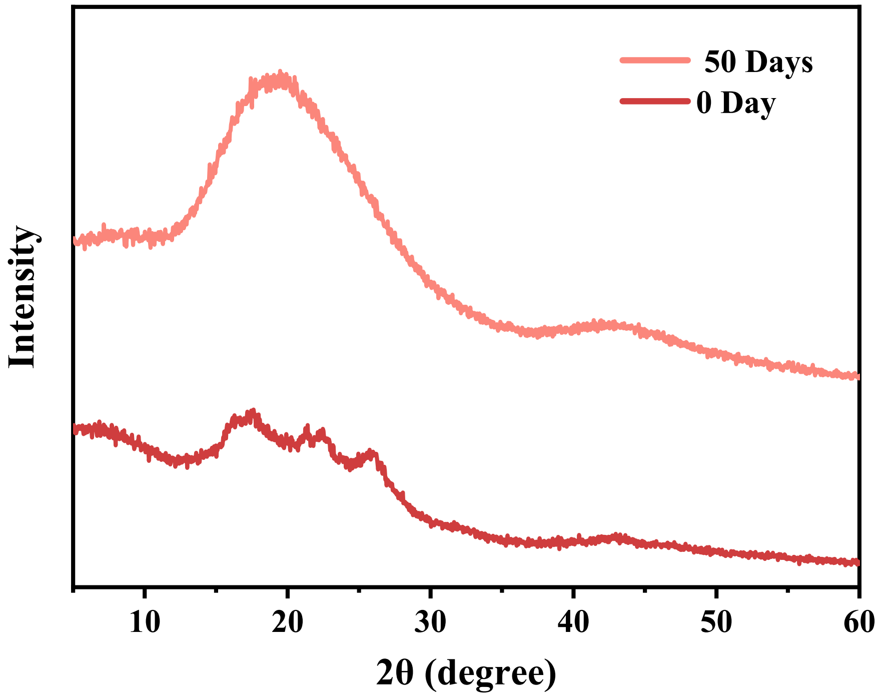

Figure S10. The wide angle X-ray diffraction patterns of PEIT-20 after degradation in 0.1M NaOH solution for 0 and 50 days
